# Evaluation of Bioactive Effects of Five Plant Extracts with Different Phenolic Compositions against Different Therapeutic Targets

**DOI:** 10.3390/antiox13020217

**Published:** 2024-02-08

**Authors:** María del Carmen Villegas-Aguilar, Noelia Sánchez-Marzo, Álvaro Fernández-Ochoa, Carmen Del Río, Joan Montaner, Vicente Micol, María Herranz-López, Enrique Barrajón-Catalán, David Arráez-Román, María de la Luz Cádiz-Gurrea, Antonio Segura-Carretero

**Affiliations:** 1 Department of Analytical Chemistry, University of Granada, 18071 Granada, Spain; marivillegas@ugr.es (M.d.C.V.-A.); alvaroferochoa@ugr.es (Á.F.-O.); darraez@ugr.es (D.A.-R.); ansegura@ugr.es (A.S.-C.); 2 Institute of Research, Development and Innovation in Biotechnology of Elche (IDiBE) Miguel Hernández University (UMH), 03202 Elche, Spain; n.sanchez@umh.es (N.S.-M.); vmicol@umh.es (V.M.); mherranz@umh.es (M.H.-L.); e.barrajon@umh.es (E.B.-C.); 3 Institute of Biomedicine of Seville (IBiS), Hospital Universitario Virgen del Rocío, CSIC, Universidad de Sevilla, 41013 Seville, Spain; cdelriomercado@gmail.com (C.D.R.); jmontaner-ibis@us.es (J.M.); 4 Department of Neurology, Hospital Universitario Virgen Macarena, 41009 Seville, Spain; 5 CIBEROBN (Physiopathology of Obesity and Nutrition CB12/03/30038), Carlos III Health Institute, 28029 Madrid, Spain

**Keywords:** phenolic compound, HPLC-MS, free radical scavenging capacity, enzyme inhibition, antioxidant, neuroprotection, structure–activity relationship

## Abstract

Plant extracts rich in phenolic compounds have been reported to exert different bioactive properties. Despite the fact that there are plant extracts with completely different phenolic compositions, many of them have been reported to have similar beneficial properties. Thus, the structure–bioactivity relationship mechanisms are not yet known in detail for specific classes of phenolic compounds. In this context, this work aims to demonstrate the relationship of extracts with different phenolic compositions versus different bioactive targets. For this purpose, five plant matrices (*Theobroma cacao*, *Hibiscus sabdariffa*, *Silybum marianum*, *Lippia citriodora*, and *Olea europaea*) were selected to cover different phenolic compositions, which were confirmed by the phytochemical characterization analysis performed by HPLC-ESI-qTOF-MS. The bioactive targets evaluated were the antioxidant potential, the free radical scavenging potential, and the inhibitory capacity of different enzymes involved in inflammatory processes, skin aging, and neuroprotection. The results showed that despite the different phenolic compositions of the five matrices, they all showed a bioactive positive effect in most of the evaluated assays. In particular, matrices with very different phenolic contents, such as *T. cacao* and *S. marianum,* exerted a similar inhibitory power in enzymes involved in inflammatory processes and skin aging. It should also be noted that *H. sabdariffa* and *T. cacao* extracts had a low phenolic content but nevertheless stood out for their bioactive antioxidant and anti-radical capacity. Hence, this research highlights the shared bioactive properties among phenolic compounds found in diverse matrices. The abundance of different phenolic compound families highlights their elevated bioactivity against diverse biological targets.

## 1. Introduction

Phenolic compounds are a class of compounds present in plants that have been reported to have enormous bioactive potential [1]. These compounds are secondary metabolites naturally synthesized in plants, and more than 8000 different chemical structures have been reported [2]. Based on their basic chemical structures, these compounds have been classified into at least 10 different classes [3]. It should be noted that, even though they show so much structural variability, they are widely studied for their antioxidant [4,5], anti-inflammatory [6], anti-aging [7], neuroprotective [8], and anticarcinogenic [9] potentials, among others. In fact, plant sources with different phenolic contents have been demonstrated to be involved in multiple pathways in the pathogenesis of different diseases [10,11]. In this scenario, there is emerging evidence on the neuroprotective activity of different phenolic compounds present in various plant sources regarding inhibition of acetylcholinesterase (AchE), Aβ aggregation, proinflammatory markers, and 1-methyl-4-phenyl-1-1,2,3,6-tetrahydropyridine (MPTP)-induced apoptosis, among others [12]. Thus, phenolic compounds from different matrices have been shown to have neuroprotective capacity, such as *Eucommia ulmoides* Oliver (rich in phenolic acids, flavonoids, and iridoid glycosides), herbaceous peony (with paeoninflorin as the main active ingredient), and *Sanghuangprous vaninii* (an extract rich in caffeic acid); they may ameliorate the dopaminergic deficiency in the MPTP-induced model in the zebrafish brain [12,13,14]. However, common phenolics present in different sources, for example, quercetin, which is contained in different matrices such as apple, chocolate, or cherry [15], have been shown to have both antioxidant and tyrosinase inhibitory actions [16]. Another example is kaempferol, another flavonoid, which is also present in a wide variety of plants and has a high antioxidant capacity [17]. When compared to other groups—for example, verbascoside, which belongs to the phenylpropanoids and is present in different matrices such as *Lippia citriodora* and *Olea europaea*—they have also been shown to have a high antioxidant capacity [18]. In this context, the structure–bioactivity relationship mechanisms are not yet known in detail for specific classes of phenolic compounds present in different plant sources.

Hence, the aim of this study is to evaluate the bioactive potential of different plant extracts with different phenolic profiles and to analyse the structure–bioactivity relationship mechanisms of phenolic compounds present in different plant sources. For this purpose, five plant matrices (fruits of *Theobroma cacao* (TC), calyxes of *Hibiscus sabdariffa* (HS), fruits of *Silybum marianum* (SM), leaves of *L. citriodora* (LC), and leaves of *O. europaea* (OE)) were selected. On these selected plants, a hydroalcoholic mixture extraction was carried out, and in order to know the phenolic content, a characterization by HPLC-ESI-TOF-MS was performed.

## 2. Materials and Methods

### 2.1. Chemicals

All chemicals used in this study were of analytical reagent grade and used as received. LC-MS grade acetonitrile and formic acid for mobile phases were purchased from Riedel-de-Haën (Honeywell, Charlotte, NC, USA). For solutions, ultrapure water was obtained with a Milli-Q system Millipore (Bedford, MA, USA), and absolute ethanol was purchased from VWR chemicals (Radnor, PA, USA). The chemical standards (verbascoside, myrecetin-3-glucoside, quercetin, epigallocatechin, gardoside, sylibin, oleuropein, kaempferol, catechin, quercetin glucoside, quinic acid, and procyanidin B1) were obtained from Sigma-Aldrich (St. Louis, MO, USA).

The following reagents were used for the different in vitro assays—sulfuric acid and absolute ethanol—they were obtained from Riedel-de-Haën (Honeywell, NC, USA). Acetic acid, sodium carbonate, sodium hydroxide, TPTZ (2,4,6-tris(2-pyridyl)-s-triazine and hydrochloridic acid were purchased from Fluka (Honeywell, NC, USA). Folin reagent, gallic acid (GA), sodium phosphate monobasic and dibasic, potassium persulfate, ABTS (2,2-azinobis (3-ethylbenzothiazoline-6-sulphonate)), fluorescein, Trolox (6-hydroxy-2,5,7,8-tetramethylchroman-2-carboxylic acid), Tris (tri(hydroxymethyl)aminomethane), sodium acetate, heptahydrate ferrous sulphate, ferric chloride, DMF (dimethylformamide), NADH (β-nicotinamide adenine dinucleotide), DAF-2 (diaminofluorescein diacetate), DHR (dihydrorhodamine), potassium dihydrogen phosphate anhydrous, NBT (nitrotetrazolium blue chloride), Cayman’s xanthine oxidase fluorometric assay kit, tyrosinase inhibitor screening kit (colorimetric), acetylcholinesterase from Electrophorus, acetylthiocholine iodide, neutrophil elastase colorimetric kit, 1-10 phenantroline, sodium chloride, hyaluronidase from sheep testes, hyaluronic acid, FALGPA (*N*-[3-(2-furyl)acryloyl]-L-leucyl-glycyl-L-prolyl-L-alanine), and collagenase from *Clostridium histolyticum* were purchased from Sigma-Aldrich (St. Louis, MO, USA). NOC-5 was purchased from Chemcruz (Santa Cruz Biotechnology, Dallas, TX, USA).

Human keratinocytes (the spontaneously immortalized cell line HaCaT) were obtained from Cell Lines Service (CLS) GmbH (Eppelheim, Germany). Dulbecco’s Modified Eagle’s Medium (DMEM), fetal bovine serum (FBS), and penicillin–streptomycin solution (10,000 U/mL penicillin and 10 mg/mL streptomycin in citrate buffer) were purchased from Gibco™/Thermo Fisher Scientific (Waltham, MA, USA). Hoechst 33342 and 2′,7′-dichlorodihydrofluorescein diacetate (H2DCF-DA) probes were acquired from Molecular Probes™ (Invitrogen™/Thermo Fisher Scientific, Waltham, MA, USA).

### 2.2. Extraction Procedure from Plant Matrices

The pre-industrial extracts of the selected plant matrices were provided by NATAC Biotech S.L. (Cáceres, Spain). These extracts were obtained by ethanol/water mixture extraction, which is considered a favorable solvent for the extraction of polar substances such as phenolic compounds, in addition to being environmentally friendly and non-toxic to humans (GRAS (Generally Recognized As Safe) solvent).

The extraction parameters were optimized for each plant matrix individually and are described below. For all extracts, a solid–liquid extraction (maceration) was carried out using a hydroalcoholic mixture for two hours. A mixture of EtOH:H_2_O (80:20; *v*:*v*) was used for all samples except for SM, which was EtOH 96%. The extraction temperatures were set at 45 °C for the OE and HS extracts and 55 °C for the rest of the extracts. A solvent:plant ratio of 20:1 was used for all extracts except for SM, which was 25:1. The obtained extracts were dried using a vacuum drying, stored at room temperature, and protected from light until their analysis. For the different analytical and bioactive analyses, the extracts were reconstituted with the same solvents and mixture used for the extraction procedure.

### 2.3. HPLC-ESI-TOF-MS Analysis

TC, HS, SM, LC, and OE extracts at 5000 mg/L were analysed by high-performance liquid chromatography (Agilent 1290 HPLC, Agilent Technologies, Palo Alto, CA, USA) coupled to mass spectrometry with a quadrupole time-of-flight analyser (Agilent 6545 QTOF Ultra High Definition, Agilent Technologies, Palo Alto, CA, USA). Chromatographic analysis was carried out in reversed phase with a C18 ACQUITY UPLC BEH column (1.7 µm, 2.1 mm, 150 mm, 130 Å, Waters Corporation, Milford, MA, USA). The working temperature of the column was 60 °C. The mobile phases were (A) acidified water with 0.1% of formic acid (*v*/*v*) and (B) acetonitrile. The following mobile phase gradient was used for optimal separation: 0.00 min [A:B 100/0], 5 min [A:B 90/10], 18 min [A:B 15/85], 24 min [A:B 0/100], 25.50 min [A:B 0/100], 26.50 min [A:B 95/5], and 32.50 min [A:B 95/5]. A mobile phase flow rate of 0.4 mL/min and an injection volume of 5 µL was used.

MS acquisition was performed in electrospray negative ionization (ESI) mode in a mass range between 50 and 1200 *m*/*z*. Other parameters were as follows: gas flow rate 10 L/min; gas temperature 200 °C; nebulizer 20 psig, enveloping gas temperature 350 °C, enveloping gas flow rate 12 L/min, VCap 4000 V, nozzle voltage 500 V.

Finally, the acquired data were processed through Qualitative Analysis of MassHunter workstation software version B.06.00 (Agilent Technologies), Mzmine 2.53, and Sirius 2.0. The compounds were annotated by comparison of the MS/MS spectra with those from analytical standards or published in the literature and databases, such as SciFinder^®^, CEU Mass Mediator, Human Metabolome Data Base (HMDB), and Kyoto Encyclopedia of Genes and Genomes (KEGG).

### 2.4. In Vitro Assays for Bioactive Determination of Phenolic Compounds in Extracts

The assays described below were adapted to a 96-well polystyrene microplate, and absorbance and fluorescence measurements were performed on a Synergy H1 Monochromator-Based Multimode Microplate reader (Bio-Tek Instruments Inc., Winooski, VT, USA).

#### 2.4.1. Total Phenolic Content and Antioxidant Capacity Measurements

Total phenolic content (TPC) was measured by the Folin–Ciocalteu method using gallic acid as a reference compound for the standard curve [19]. Measurements were carried out in triplicate.

The FRAP assay was performed following the method described by Benzie and Strain (1996) [20]. The reduction of the radical cation of ABTS was performed by TEAC assay using a method previously described by Zulueta et al. (2022) [21]. To test the ability of the extracts to scavenge peroxyl radicals, an ORAC method was used with some modifications [21]. In all assays, measurements were performed in triplicate.

#### 2.4.2. Evaluation of Free Radical and ROS/RNS Scavenging Potential

All free-radical scavenging assays were performed and adapted according to Rojas-García et al. (2022) [22]. To measure the scavenging capacity of the radical superoxide anion (·O_2_^−^), a colorimetric method was used. A fluorometric-based method was used for the nitric oxide (·NO) and HOCl assays. The results for the three assays were expressed as the concentration of the different extracts needed to inhibit ROS/RNS formation by half (IC_50_).

#### 2.4.3. Evaluation of Enzymatic Inhibition Potential

The activity of the extracts to inhibit the enzyme tyrosinase was performed using the Tyrosinase Inhibitor Screening Kit (Colorimetric). The Xanthine Oxidase (XO) inhibitory activity of the extracts was measured using the Cayman’s XO Fluorometric Assay Kit. The elastase inhibition assay was measured using the Neutrophil Elastase Inhibitor Screening Kit. For the measurement of hyaluronidase inhibition, the test performed by Nema et al. (2013) [23] was used with some modifications. The inhibitory effect against collagenase of the extracts was performed following the methodology performed by Kumar et al. (2019) [24], but some parameters were modified. Finally, acetylcholinesterase (AchE) inhibitory activity was measured using a photometric assay described by Ellman et al. (1961) [25]. All assays were performed in triplicate, and the result was expressed by calculating the IC_50_ using different concentrations of the extracts except for the tyrosinase and elastase assays, where the % inhibition of the enzymes was calculated at 500 and 1000 mg/L concentration of the extracts, respectively.

#### 2.4.4. Cellular Assays to Measure Antioxidant Capacity

##### Cytotoxic Activity In Vitro

The cytotoxic activity of the extracts was assessed on human immortalized keratinocytes (HaCaT cell line). Cells were maintained following the manufacturer’s indications. DMEM with 4.5 g/L glucose and 1 mM pyruvate was supplemented with 10% (*v*/*v*) of fetal bovine serum and 1% (*v*/*v*) penicillin–streptomycin solution. Cells were grown at 37 °C in a humidified 5% CO_2_ incubator and were passed every 2–3 days. Assays were carried out in 96-well plates with 12,000 seeded cells in each well. 

Cells were seeded and were treated after 24 h with each extract at different concentrations (5–800 µg/mL). Cells were incubated for 24 h, and nuclei were stained by adding Hoechst 33342 fluorescent probe during the last 30 min (4.5 µM final concentration). Extracts were freshly prepared at 100 mg/mL in DMSO, and corresponding DMSO controls were included in the assay to evaluate cytotoxic effects caused by this solvent. 

Fluorescence measurements were carried out in PBS 1x using a Cytation 3 Cell Imaging Multimode reader (BioTek, Winooski, VT, USA) with 377 nm excitation and 447 nm emission filters. Data were expressed as a percentage of cellular viability compared to nontreated cells. IC_50_ values were calculated through nonlinear regression of the algorithm-transformed concentrations and the normalized responses.

##### Antioxidant Activity In Vitro

To evaluate the antioxidant effectiveness of the extracts, HaCaT cells were cultured for 24 h as described above and then treated with noncytotoxic concentrations (10, 20 y 40 µg/mL) of the extracts. After 24 h of treatment, cultures were washed twice with PBS 1x. Cells were maintained with a thin layer of PBS 1x while were exposed to solar ultraviolet radiation type A (UVA) radiation (8 J/cm^2^) emitted by a Bio-Link Crosslinker BLX-E312 (Vilber Lourmat, Collégien, France). To prevent excessive heating due to the UVA exposure, plates were meanwhile put on ice as described previously by Cooper et al. (2009) [26]. In parallel, treated cells were manipulated in the same manner but were covered during UVA exposure (nonirradiated controls). Subsequently, cells were incubated with Hoechst 33342 (4.5 µM) and H2DCF-DA (30 µM) in fresh medium for 30 min. H2DCF-DA (nonfluorescent) to monitor ROS generation through its oxidation to fluorescent 2′,7′-dichlorodihydrofluorescein (DCF) by those radical species and cell viability (Hoechst). Both fluorescent signals were measured by the Cytation 3 reader as described above for Hoechst 33342 and using 485 nm excitation and 535 nm emission filters for DCF. DCF signals were normalized with the nuclei number determined for each well by Hoechst staining, and data were expressed as percentage of ROS (%) compared to nontreated and nonirradiated cells.

### 2.5. In Vivo Neuroprotection Capacity

#### 2.5.1. Drosophila Stock and Exposure to Hypoxia

*Drosophila melanogaster* has been commonly employed as a neurodegeneration model system due to its minimal resource demands and notable conservation, including the response to alterations in oxygen levels, which is similar to the human. Hypoxia-induced injury is a crucial mechanism in several medical conditions, including ischemic stroke, cardiac infarction, and renal disease, among others. In fact, drosophila has been proposed by several authors to study the impact of hypoxia-reperfusion [27,28] and represents a good screening model for neurovascular disease [29]. 

*Drosophila melanogaster* stock (Oregon R strain) was kindly provided by Dr. Luisma Escudero. Flies were bred in polystyrene tubes on a standard medium at constant temperature and humidity (25 °C; 50% humidity) and a 12 h light/dark cycle. Three days after emergence, male flies were sexed under CO_2_ anesthesia, and vials containing 10–15 male flies were prepared. Treated flies received instant food formulation (Genesee Scientific, Morrisville, NC, USA) prepared in water containing the plant extracts at the indicated concentrations. After hatching, male flies were maintained in standard food for 5 days, and a set of flies were supplemented in the food with the plant extracts at 0.05, 0.2, and 0.3 mg/mL. The control group was kept on media prepared in vehicle (0.5% ethanol in water). Treatment media was refreshed once during the experimental procedure. After 5 days, flies were subjected to 2.5 h of hypoxia (1% O_2_, 25 °C, 30–40% humidity) by introducing the vials in a hypoxic glove box (Coy, Grass Lake, MI, USA) where the environmental oxygen was displaced by N_2_. Then, flies were monitored for locomotor activity for 4 h and mortality rate was assessed after reoxygenation and represented as relative mortality to the hypoxia group. Each experiment consisted of three tubes per condition (10–15 flies/vial) and was repeated at least three times.

#### 2.5.2. Drosophila Locomotor Activity Monitoring

To study the effect of hypoxia exposure on fly behavior, flies were transferred into a 25 mm empty polycarbonate tube and placed in the Drosophila Activity Monitoring (DAM) system v3.11.1.35 (LAM25H-3, Trikinetics Inc., Waltham, MA, USA). Locomotor activity was recorded for 4 h by registering the infrared light beam crosses in each tube at 3 different heights. The DAMSystem3 Data Collection Software was used for data acquisition, and raw data were grouped into 30-minute intervals using FileScan Software v1.13. Live flies were counted at the beginning and the end of the assessment period. Relative mean beam crosses were calculated by normalizing mean movement counts per fly to the hypoxia group and represented as a heatmap using GraphPad Prism v7.

### 2.6. Statistical Analysis

The results were presented as mean ± standard deviation (SD) of at least three replicates. IBM SPSS Statistics 24.0 software (SPSS Inc., Chicago, IL, USA) was used for the statistical analysis of antioxidant, free radical, and enzyme data. The differences between samples were statistically analysed by one-way ANOVA, and post hoc comparisons of the means were performed with Tukey’s HSD and T3 de Dunnett tests. 

GraphPad Prism version 8.01 (GraphPad Software, San Diego, CA, USA) was used for the representation and analysis of cellular assays. Data were expressed as the mean ± SD of 5–10 replicates depending on the assay. Statistical differences were determined by one-way ANOVA and statistical comparisons with Tukey’s test.

## 3. Results

### 3.1. Characterisation of the Extracts by HPLC-ESI-qTOF-MS

The TC, HS, SM, LC, and OE extracts were tentatively characterized by HPLC-ESI-qTOF-MS. Base peak chromatograms (BPCs) of the five extracts are shown in Figure S1. The characterization was carried out based on retention times, fragments, mass spectra, predictions from different software, and other studies previously published in the literature. According to the identification guidelines proposed by Sumner et al. (2007) [30], compounds were annotated at level 1 with commercial standards, at level 2 by comparing the MS/MS spectra with those present in the databases, at level 3 based on the molecular formulation and MS1 spectra, and at level 4 where the molecules remain as unknowns. All this information is provided in Tables S1, S2, S3, S4, and S5 for TC, HS, SM, LC, and OE extracts, respectively.

In total, 292 compounds were characterized, specifically 52 compounds in TC, 40 compounds in HS, 67 compounds in SM, 85 compounds in LC, and 98 compounds in OE. It was worth noting the difference between the matrices in phenolic richness, with SM, LC, and OE having more than 65 compounds in each, while TC and HS have less than 60 compounds. This fact may be mainly due to the extraction conditions and the polarity of the majority of phenolic compounds in each matrix. Among the five extracts, LC and OE contain the highest number of annotated compounds. Briefly, the flavan-3-ols was the class with the highest richness in the TC extract since epigallocatechin, quinic acid, and gluconic acid were the main compounds present in this extract. The HS extract has a high presence of hibiscus acid, hibiscus acid lactone, and glycosylated flavonoids, such as quercetin 3-O-rutinoside and quercetin 7-glucoside. The SM extract stood out for its high flavonoid presence, especially silybin and its isomers such as silycystin, isosilybin b, or its modified forms such as dehydrosilybin, silybin hydrogenated or acetylsilybin A/B. The LC extract was characterized by a particularly high presence of phenylpropanoids. Among the phenylpropanoids, verbascoside presented the highest presence. In addition, a high presence of iridoids and secoiridoids, such as shanziside, and glycosylated compounds of this type, such as gardoside, was detected. In the OE extract, the presence of the oleuropein aglycone was particularly high. The parental form of this compound, oleuropein, and other modifications of oleuropein, such as oleuropein-glucoside, were also found in high concentrations.

Table 1 shows the common compounds among the five matrices under study. The high presence of fatty acids shared by the five matrices is noteworthy. The matrices with the highest number of compounds in common were LC and OE, with verbascoside, malic acid, gluconic acid, and fatty acids such as linolenic acid and palmitic acid standing out among the compounds in common.

### 3.2. Evaluation of the Antioxidant and Anti-Inflammatory Capacities of the Extracts

#### 3.2.1. Evaluation of TPC, Antioxidant Capacity and ROS Scavenging Potential

In Table 2, we can find the TPC values obtained by the Folin–Ciocalteu method and the results of the FRAP, TEAC, and ORAC tests for the five matrices under study. As a result, all the matrices under study show antioxidant capacity, so they can all be considered bioactive against oxidative stress. It is worthwhile to highlight the case of the SM extract, which obtained the highest values for the four tests. In addition, a general trend showing that the higher the content of TPC, the greater the capacity for the transfer of electrons and H atoms can be observed, confirming previous studies [31]. This is also in agreement with the findings in the case of the HS extract, which presented the lowest values in the four assays. It is important to note that there is no universal method for measuring antioxidant capacity, as different methods can measure various mechanisms of action. For instance, assays like FRAP and TEAC are based on single electron transfer (SET), employing indirect and direct approaches, respectively. On the other hand, the ORAC method relies on hydrogen atom transfer (HAT), which is similar to both electron transfer and hydrogen atom transfer. Choosing different assays to evaluate antioxidant activity can offer a comprehensive prediction of this bioactive potential, providing complementary information [32].

Table 3 reveals the amount of extract required to inhibit half the concentration of reactive species (IC_50_).

The intracellular accumulation of ROS, which occurs in cells under oxidative stress, is responsible for several chronic pathologies, including cancer, neurodegenerative or cardiovascular pathologies [33]. Thus, Reuter et al. (2010) revealed that oxidative stress can activate several transcription factors, which can lead to the expression of more than 500 different inflammation-related genes [34]. The activation of this entire cascade can lead to chronic inflammation, which in turn may mediate most chronic diseases, including cancer, diabetes, cardiovascular, neurological, and pulmonary diseases [34]. In this regard, phenolic compounds have been shown to have a potent antioxidant effect because their chemical structure means they can eliminate ROS, and their antioxidant capacity is therefore related to the other properties of this type of compound, such as anti-inflammatory and neuroprotective properties.

In relation to radical scavenging assays, with the exception of LC and OE for the ·O_2_^−^ assay, all showed bioactivity. TC presented the best IC_50_ value for all the radical scavenging tests, being significantly better than the other extracts. This indicates that the types of phenolic compounds present in TC have a higher anti-radical power than those present in OE for the ·O_2_^−^ and HOCl test and that the HS for the ·NO test presented the highest IC_50_ values. For these tests, epicatechin (EPI) and gallic acid (GA) were used as standard controls, listed in Table 3. It is observed that, especially in the HOCl assay, EPI has significantly better values compared to the five plant matrices, which is related to the TC extract being one of the lowest as this extract has a high EPI content.

#### 3.2.2. Evaluation of Enzymatic Inhibition Capacity

Table 4 presents the inhibitory effect of the five extracts under study on the hyaluronidase, XOD, tyrosinase, elastase, and collagenase enzymes and the positive control used for each enzyme. As mentioned above, phenolic compounds are involved in the regulation of the level of reactive species. In this sense, the excess of these reactive species can lead to excessive activation and dysregulation of different enzymes studied in this work. For instance, the enzyme XOD is a dehydrogenase responsible for catalyzing hypoxanthine to xanthine and subsequently to the oxidation of uric acid. However, when oxidative stress is present, XOD is transformed into an oxidase, which leads to the production of superoxide radicals and causes many inflammatory diseases [35].

In the case of the hyaluronidase assay, all matrices under study show bioactivity. Nevertheless, SM has been shown to be the most bioactive, while OE is significantly less so. For the enzyme XOD, the lowest IC_50_ value corresponds to OE, while TC and HS did not even reach this 50% inhibition. Furthermore, when compared to the positive control used, EPI, both SM and LC, and OE have a more significant ability to inhibit the enzyme. The tyrosinase inhibition assay shows the % inhibition of the enzyme at 500 mg/L, with SM showing the highest activity and HS the lowest and with the other three extracts being similar in terms of inhibition. In the elastase assay, the results were expressed as % inhibition of the enzyme at 1000 mg/L, with the LC extract having the highest inhibitory power, while SM is not able to inhibit. Thus, in this case, with the exception of SM, all the extracts showed bioactivity. Finally, in the collagenase assay, the five matrices under study were able to inhibit collagenase, highlighting, in this case, SM since, when compared to HS, which is the least bioactive, there is a big difference in the dose needed to inhibit the enzyme at 50%.

#### 3.2.3. Cellular Assays to Measure Antioxidant Capacity

**Figure 1:**
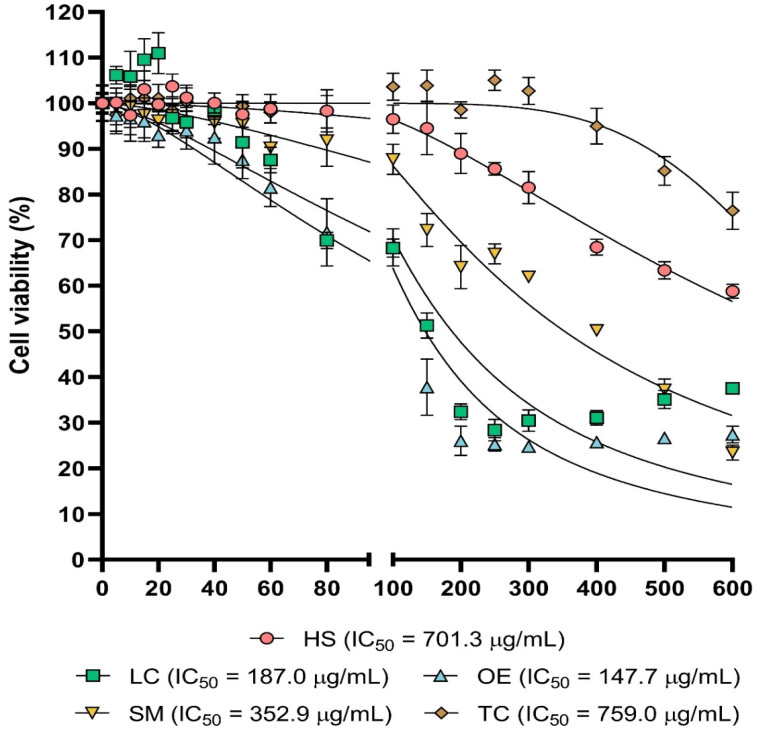
Cytotoxic activity. Dose–response cell viability plots for the five extracts tested. Cell viability values were obtained as described in methods section. Statistical significance was included in Table S6.

The cytotoxic effects of the extracts in HaCaT cells were plotted in Figure 1 (Statistical significance for Figure 1 was included in Table S6). DMSO was used as a vehicle to solubilize the extracts and not alter cell viability, even at the highest used concentration (Table S7). 

LC and OE extracts exhibited the highest cytotoxicity, and both treatments resulted in a statistically significant reduction in cell viability from 40 µg/mL. IC_50_ was estimated as 187 µg/mL for LC and 147.4 µg/mL for OE. In the case of the SM extract, cytotoxic effects were statistically significant from 60 µg/mL, and IC_50_ was 352.9 µg/mL. With the HS treatment, cell viability was decreased from 200 µg/mL with statistical significance, and calculated IC_50_ was 701.3 µg/mL for this extract, which did not reduce cell viability in a significant manner up to 400 µg/mL and whose IC_50_ was 759 µg/mL.

**Figure 2:**
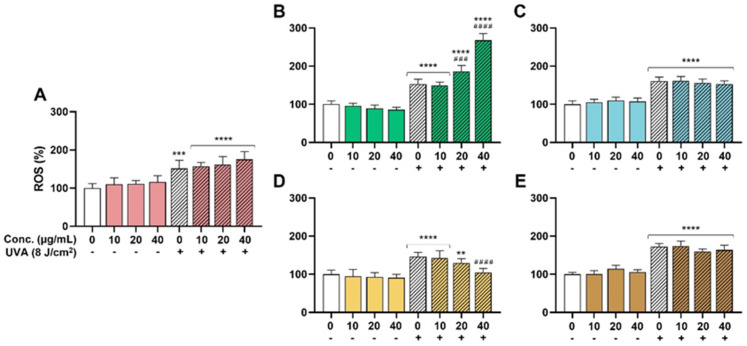
Antioxidant activity. ROS levels were measured as described in methods section. Significance was established at *p* < 0.05. ** (*p* < 0.01), *** (*p* < 0.001), and **** (*p* < 0.0001) are in the figures and indicate statistically significant differences compared to the nontreated and nonirradiated control. ### (*p* < 0.001), and #### (*p* < 0.0001) indicate statistically significant differences compared to the nontreated irradiated control. (**A**): *Hibiscus sabdariffa*; (**B**): *Lippia citriodora*; (**C**): *Olea europaea*; (**D**): *Silybum marianum*; (**E**): *Theobroma cacao*.

The effectiveness of the extracts as antioxidant ingredients was explored in the HaCaT model due to the significant role of oxidative stress on skin health and aging. UVA was chosen as a well-known inductor of ROS generation and oxidative stress in the skin [36]. On the one hand, as shown in Figure 2, none of the extracts were able to reduce the basal oxidative stress in the absence of UVA. On the other hand, UVA radiation increased the presence of ROS in a significant way in all the conditions, and only the SM extract was able to decrease these ROS levels in a dose-dependent manner. Pretreatment with 20 µg/mL of SM reduced the increment in ROS levels from 146% (untreated but irradiated condition) to 129%. A statistically significant reduction to 105% ROS was evidenced for 40 µg/mL (Figure 2D, ####, *p* < 0.0001). Apparently, the rest of the extracts (HS, OE, LC, and TC) did not prevent the oxidative action of UVA. Furthermore, the LC extract exhibited a significant prooxidant effect at 20 and 40 µg/mL, probably related to a phototoxic effect of some of its components.

### 3.3. Evaluation of the Neuroprotective Effect of the Extracts

There is a close link between the ability of phenolic compounds to exert their neuroprotective effect through their antioxidant and free radical scavenging action and their ability to inhibit enzymes involved in neurodegenerative diseases, such as AChE [8].

#### 3.3.1. Evaluation of Acetylcholinesterase (AChE) Inhibition Capacity

Table 4 shows the inhibitory effect of the extracts on AChE, showing that the lowest extract concentration to inhibit 50% of the enzymatic activity was for the TC extract, followed by LC, OE, and SM extracts. The HS extract did not even reach 50% of the enzymatic activity at very high doses of concentration. Thus, with the exception of HS, all showed bioactivity against enzyme inhibition.

#### 3.3.2. Effect of Supplementation with Different Plant Extracts on Hypoxia–Reoxygenation Injury in *D. melanogaster*

**Figure 3:**
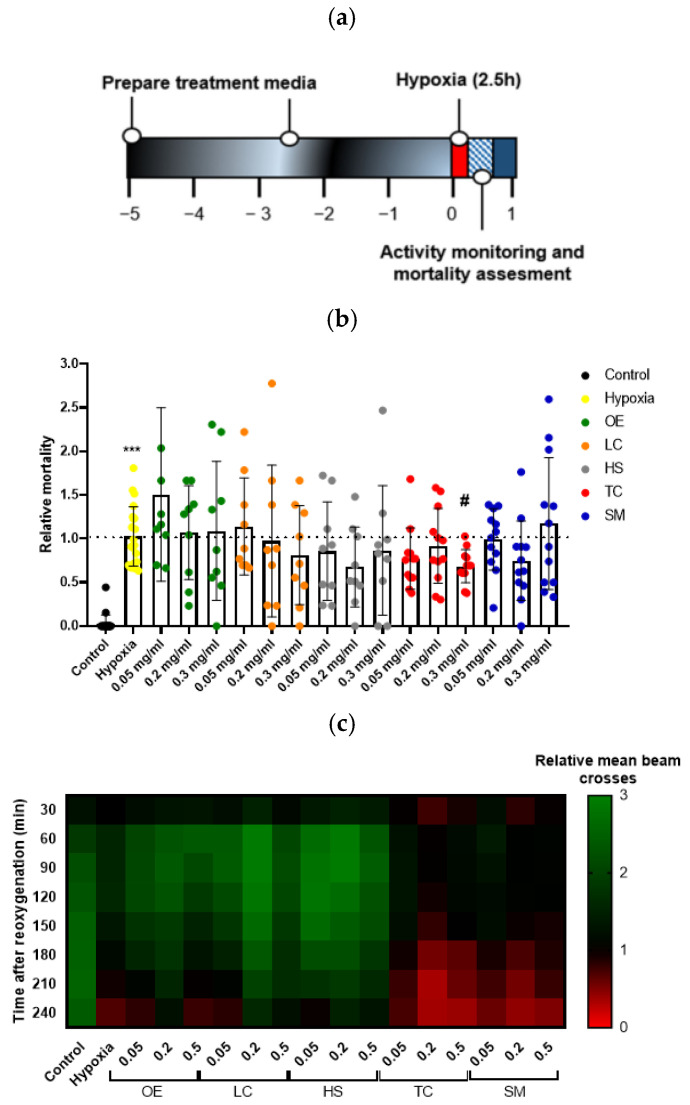
Effect of plant extract supplementation on Drosophila melanogaster exposed to severe hypoxia. (**a**) Schematic illustration of the hypoxia protocol in flies. (**b**) Effect of the treatment on fly survival after exposure to 1% O_2_ for 2.5 h. Data were analyzed using one-way ANOVA test followed by Dunnett´s multiple comparisons test *** *p* < 0.001 vs. control; # *p* < 0.05 vs. hypoxia. (**c**) Heatmap displaying the locomotor activity of treated flies. Flies were transferred into the monitor immediately after hypoxia and the activity was recorded for 240 min after reperfusion. Each cell shows the mean beam crosses per fly in the group.

Hypoxic stress is known to produce injury in flies. The drosophila model does not reproduce some aspects of human brain ischemia because of its primitive blood system (blood vessels are lacking, and there is no lymphoid blood cell lineage). However, the model recapitulates important pathogenic features when subjected to hypoxia, such as increased activation of brain caspases, locomotor deficiencies, and mortality [37]. Moreover, increases in several oxidative stress markers and changes in metabolic activity have been observed in flies subjected to hypoxia [27]. Therefore, the use of this screening model provides an easy and convenient way of testing antioxidant compounds, such as polyphenols. To investigate the influence of hypoxia–reoxygenation injury in Drosophila, treated flies were subjected to hypoxic stress for 2.5 h under controlled conditions (1% O_2_; 25 °C; 30–40% relative humidity) (Figure 3a). As expected, flies in the hypoxia group showed a significant increase in mortality compared to the control group 4 h after reoxygenation. Treatment with the extract TC at the higher concentration resulted in a significant increase in fly survival after the hypoxia challenge (Figure 3b). 

We also studied fly behavior by quantifying animal movement for 4 h after hypoxia. While control flies moved uniformly over time, flies in the hypoxia group showed a reduction in locomotor activity, indicated by fewer beam crosses, which were more evident after 120–150 min of reoxygenation, revealing that the reperfusion injury worsened fly behavior. However, treatment with TC could not recover the loss of locomotor activity induced by hypoxia (Figure 3c).

The results obtained show that there is no single matrix that stands out for its bioactivity in all the tests, but rather, depending on the bioactive target and the assay in question, there are matrices that stand out. Still, the rest, with a few exceptions, also show bioactivity. This shows that despite the structural diversity present in the different families of phenolic compounds, they all show high bioactivity; it is the combination of these compounds that gives them their pleiotropic character.

## 4. Discussion

The extracts under study have been characterized, giving a wide range of phenolic compounds, some of which are characteristic of each of the matrices and a minority of which are common among the different matrices (Table 1). Although most of the compounds are not common, all matrices have bioactive potential against the targets under study, so the differences in potential between them may be mainly due to these specific compounds being in each of the matrices and also due to the differences in the ratio between them.

When we pay attention to the results of the antioxidant tests for TPC and FRAP, TEAC, and ORAC (Table 2), the higher the TPC, the greater the antioxidant power shown in the tests. Thus, HS has the lowest TPC and the lowest value in the other antioxidant test values, while SM has the highest TPC content and high values in the rest of the tests measuring antioxidant power. This relationship is consistent with the tests carried out by Aroso et al. (2017) [38], in which there is also a positive relationship between the TPC content and the tests measuring antioxidant power.

In the case of the other three remaining plant matrices, they have intermediate values for the results of the TPC and FRAP, TEAC, and ORAC assays. For the FRAP assay, the matrices TC, SM, and OE showed very similar values. This may be due to the fact that there are similar compounds in their composition. For example, both TC and OE have epigallocatechin [39], a compound that has been shown to have a potent action in the FRAP assay. In contrast, SM, with its high content of flavolignans such as silybin A and B, has also been shown to have a potent action in the FRAP assay [40]. In the case of the ORAC assay, the matrices LC and OE obtained a similar and high value, which may be due to the fact that both matrices share the most compounds in their composition, like verbascoside, which has been shown to be potent in proton transfer, the mechanism of action on which this antioxidant capacity test is based [41].

In the case of cellular assays in HaCaT cells, TC, OE, and SM extracts were able to decrease ROS levels; however, the only one that achieved this in a dose-dependent manner was SM. This is in agreement with Svobodová et al. (2007) [42], who found that flavonolignans present in the SM extract suppress UVA-induced oxidative stress in HaCaT cells, making this extract potentially useful in the treatment of UVA-induced skin damage.

In relation to the tests to measure the capacity to eliminate specific ROS and RNS, for the ·O_2_^−^ radical test, the HS and SM matrices obtained a similar IC_50_ value, which is also similar to the value obtained for the gallic acid standard. Table 1 shows that the compounds that these two matrices have in common include chlorogenic acid and quercetin glucoside. In this sense, there are studies that demonstrate the high power of chlorogenic acid [43], quercetin glucoside [44], and gallic acid [45] for the uptake of the superoxide radical, which is in agreement with the results obtained.

In the test to eliminate HOCl, TC and SM were the ones that obtained the lowest IC50. Despite the difference in phenolic composition, they achieved similar results. TC extract has a high epigallocatechin and epicatechin content. As shown in Table 3, epicatechin has a high free radical scavenging power, with its power in the HOCl scavenging test standing out, where it has more power than even the whole TC extract. This is supported by different studies showing the high power of TC extract to scavenge free radicals [46]. Furthermore, He et al. (2018) evaluated the power of different catechins against radicals, and they found that epigallocatechin gallate possessed the highest radical scavenging power, followed by epigallocatechin, epicatechin, and catechin in descending order of power. This suggests that the effect of these compounds is strongly related to the structure of catechins, mainly due to the hydroxyl and galloyl groups [47]. For SM, silybin has shown potent action for HOCl removal [48].

In the case of the XOD enzyme, the OE and LC extracts have been shown to have potent inhibitory power. Both extracts share compounds such as verbascoside, the main bioactive compound present in LC. This compound was shown to be a potent inhibitor of the XOD enzyme, as verbascoside is able to enter the active site of XOD and form hydrogen bonds with amino acid residues (such as Lys-1045, Arg-880, Arg-912, Glu-1261, and Gln-1194) [49].

Within the enzymes involved in maintaining skin firmness, hyaluronidase, elastase, and collagenase, there is no single matrix that stands out for all of them. For both hyaluronidase and collagenase, the TC and SM matrices have the lowest IC_50_ values. Thus, in the case of TC, the content of compounds from the flavan-3-ols group was shown to be potent inhibitors of these three enzymes involved in the loss of elasticity and firmness of the skin [50]. For SM, the main group of compounds are flavolignans, which have also been shown to have a potent inhibitory action on the aforementioned enzymes. This shows that phenolic compounds from different groups and, therefore, with different chemical properties can exert the same bioactive effect [51].

The mechanism of action by which the polyphenols present in our matrices have the ability to inhibit enzymes would be the next step in our research. There are in silico studies—using different phenolic compounds that have been tested on the enzymes—for example, the inhibition of rosmarinic acid against human hyaluronidase. Molecular docking studies revealed that rosmarinic acid is bounded to the hyaluronidase binding pocket with four binding interactions [52]. In our study, the hyaluronidase used was not human but bovine, but these are highly phylogenetically conserved proteins [53]. Another compound that has been tested in silico against collagenase, elastase, and tyrosinase enzymes is caffeine, which was shown to form a stable protein–ligand complex validated by molecular dynamics simulation. Thus, the potential of phenolic compounds in the inhibitory action of these enzymes is shown [54].

Finally, the neuroprotective effect of the different extracts was evaluated in both in vitro and in vivo assays. In the case of the in vitro AChE enzyme inhibition assay, the extract with the highest enzyme inhibitory capacity was TC, followed by LC and OE extracts with a similar IC_50_ value. In the case of the in vivo assay in which the survival of flies after hypoxia challenge was measured, it was TC that significantly improved survival. The TC extract is high in epigallocatechin and epicatechin, compounds that have been shown to be potent inhibitors of AChE [55,56]. The similar inhibition of LC and OE at doses slightly higher than TC may be due to their high presence of flavonoid glycosides, which are considered essential for AChE inhibition [57].

The results highlight the importance of reporting the bioactive properties of plant extracts according to their phenolic composition and not simply their total phenolic content, as many of the bioactive properties can be related to a particular phenolic type or phenolic family or to the synergistic action of them.

In this context, when exploring the potential use of bioactive compounds, such as phenolic compounds, for health improvement, utilizing combined plant extracts becomes intriguing. A rich array of phenolic compounds from diverse sources could offer broader benefits to the organism, leveraging the potential synergistic effects arising from different types of phenolic compounds [58]. Therefore, plant extracts with this bioactive potential could be used for the development of nutraceuticals for the prevention of diseases related to metabolic stress and inflammation [59]. Additionally, these extracts could be employed in nutricosmetics, as the main enzymes evaluated—such as tyrosinase, collagenase, and hyaluronidase—are associated with maintaining skin color, elasticity, and hydration, respectively [60].

## 5. Conclusions

In this study, the antioxidant, anti-inflammatory, and neuroprotective effects of five plant extracts showing different phenolic compositions have been evaluated by means of different assays in order to assess the relationship between the presence of different types of phenolic compounds and their bioactivity. This study shows that not all extracts that are rich in phenolic compounds show the same bioactivities in the assays used, but, depending on their phenolic composition, there are extracts with greater or lesser bioactive potential against different targets. In general, it is observed that when there are common phenolic compounds in the different extracts, some of the bioactive capacities are similar, as has been observed in the case of LC and OE. Nevertheless, the greatest bioactive difference has been observed when comparing the richness of the families of phenolic compounds, so the SM extract, rich in flavolignans such as silybin, are shown to possess a high antioxidant capacity in both spectrophotometric and cellular assays. It also proved to be a potent inhibitor of tyrosinase, hyaluronidase, and collagenase. In contrast, in other bioactivity tests measuring free radical scavenging capacity and neuroprotective effect through inhibition of the enzyme AChE and survival in flies subjected to hypoxia stress, it was the TC extract that stood out, possibly due to its high presence in compounds of the flavan-3-ol family, such as epigallocatechin and epicatechin. In future studies, it would be interesting to study the relationship between the phenolic composition of the matrices and the macronutrients present in the same matrices, with the aim of evaluating whether these interactions affect the hypoactivity of the phenolic compounds. Thus, this work demonstrates that phenolic compounds present in different matrices have common bioactive properties and that the abundance of different families of phenolic compounds makes them stand out with higher bioactivity against different biological targets.

## Figures and Tables

**Table 1:** Common compounds that have been identified in more than one of the matrices.

Compound	Rt (min)	[M-H]^−^	Mol. Formula	*T. cacao*	*H. sabdariffa*	*S. marianum*	*L. citriodora*	*O. europaea*
Gluconic acid	1.01	195.0519	C_6_H_12_O_7_	x			x	x
Malic acid	1.07	133.0140	C_4_H_6_O_5_				x	x
Quinic acid	1.13	191.0292	C_7_H_12_O_6_	x				x
Chlorogenic acid	4.02	353.0867	C_16_H_18_O_9_		x	x		
Epigallocatechin	9.18	305.0690	C_15_H_14_O_7_	x				x
Verbascoside	9.33	623.1978	C_29_H_36_O_15_				x	x
Quercetin 3-O-rutinoside	9.60	609.1458	C_27_H_30_O_16_		x			x
Quercetin glucoside	9.85	463.0878	C_21_H_20_O_12_	x	x	x		
Unknown	10.23	539.1751	C_25_H_32_O_13_		x			x
Quercetin	12.16	301.0339	C_15_H_10_O_7_	x	x			
Unknown	12.95	329.0654	C_17_H_14_O_7_		x		x	
Dihydrocapsiate	13.53	307.192	C_18_H_28_O_4_				x	x
Eupatorin	13.56	343.0818	C_18_H_16_O_7_		x		x	
Gingerol	13.92	293.1748	C_17_H_26_O_4_	x	x		x	x
Hydroxylinolenic acid	15.74	293.2113	C_18_H_30_O_3_		x		x	x
Unknown	16.30	540.3288	C_29_H_49_O_9_	x		x	x	x
Unknown	16.60	566.3453	C_31_H_51_O_9_			x	x	x
Ethyl vanillate	17.77	195.0686	C_10_H_12_O_4_	x				x
Linolenic acid	18.46	277.2159	C_18_H_30_O_2_	x	x		x	x
10′-Apo-beta-carotenal	18.73	375.2712	C_27_H_36_O	x	x	x	x	x
Linoleic acid	19.16	279.2328	C_18_H_32_O_2_	x	x	x	x	x
Palmitic acid	19.82	255.2325	C_16_H_32_O_2_	x			x	x
Unkwnon	19.87	403.3052	C_22_H_44_O_6_	x	x	x		x
Oleic Acid	19.95	281.2482	C_18_H_34_O_2_	x	x	x	x	x
Unknown	20.98	383.1934	C_16_H_32_O_10_	x	x		x	x
Ginsenoside Rh2	20.98	621.4417	C_36_H_62_O_8_			x	x	

x: presence of the compound in the extract.

**Table 2:** Evaluation of total phenolic content and antioxidant capacity of extracts.

Sample	TPC (mg GAE/g DE)	FRAP (mmol Fe^2+^/g DE)	TEAC (mmol TE/g DE)	ORAC (mmol TE/g DE)
*T. cacao*	255 ± 12 ^a^	1.38 ± 0.09 ^b,c^	1.25 ± 0.08 ^a^	2.35 ± 0.09 ^c^
*H. sabdariffa*	51 ± 1 ^d^	0.47 ± 0.05 ^d^	0.24 ± 0.02 ^c^	1.16 ± 0.03 ^d^
*S. marianum*	617 ± 8 ^b^	1.4 ± 0.3 ^a,b,c,d^	1.3 ± 0.1 ^a^	11.7 ± 0.3 ^b^
*L. citriodora*	344 ± 15 ^c^	2.4 ± 0.2 ^a^	1.15 ± 0.08 ^a^	5.9 ± 0.2 ^a^
*O. europaea*	216 ± 28 ^a^	1.65 ± 0.10 ^a,b^	0.84 ± 0.05 ^b^	6.0 ± 0.2 ^a^

TPC: Total Polar Compounds; FRAP: Ferric Reducing Antioxidant Power Assay; TEAC: Trolox Equivalent Antioxidant Capacity; ORAC: Oxygen Radical Absorbance Capacity; GAE: Gallic Acid Equivalent; DE: Dry Extract; TE: Trolox Equivalent. Data are means ± standard deviation (*n* = 3). Different letters represent statistically significant differences at *p* < 0.05 level.

**Table 3:** Evaluation of radical scavenging of controls and extracts.

Sample	·O_2_^−^ (mg/L) ^1^	·NO (mg/L) ^1^	HOCl (mg/L) ^1^
*T. cacao*	29.7 ± 0.4 ^c^	0.42 ± 0.02 ^b^	0.71 ± 0.02 ^b^
*H. sabdariffa*	50 ± 2 ^a^	10.3 ± 1.0 ^c^	1.32 ± 0.04 ^d^
*S. marianum*	57 ± 6 ^a^	5.0 ± 0.8 ^a^	0.70 ± 0.01 ^b^
*L. citriodora*	n.d.	3.76 ± 0.08 ^a^	3.5 ± 0.4 ^a^
*O. europaea*	269 ± 17 ^d^	3.0 ± 0.2 ^a^	16 ± 2 ^c^
Gallic acid	50 ± 3 ^a^	1.4 ± 0.3 ^b^	3.8 ± 0.3 ^a^
Epicatechin	70 ± 5 ^b^	0.87 ± 0.02 ^b^	0.18 ± 0.01 ^e^

Data are means ± standard deviation (*n* = 3). ^1^ Inhibitory Concentration at 50%. Different letters represent statistically significant differences at *p* < 0.05 level. n.d.: no data.

**Table 4:** Evaluation of enzymatic inhibition capacity of controls and extracts.

Sample	Hyaluronidase (mg/L) ^1^	XOD (mg/L) ^1^	Tyrosinase (% inh.) ^2^	Elastase (% inh.) ^3^	Collagenase (mg/L) ^1^	AChE (mg/L) ^1^
*T. cacao*	12 ± 2 ^a^	n.d.	28 ± 8 ^a,b,c^	23 ± 4 ^b,c,d^	156 ±1 ^b^	244 ± 9 ^a^
*H. sabdariffa*	66 ± 6 ^d^	n.d.	5 ± 2 ^c^	44.1 ± 0.5 ^a,b,c,d^	1190 ± 35 ^b^	n.d.
*S. marianum*	4.9 ± 0.4 ^a^	4.4 ± 0.4 ^a^	39 ± 4 ^a^	n.d.	56 ± 3 ^b^	1259 ± 53 ^b^
*L. citriodora*	87 ± 5 ^c^	3.2 ± 0.4 ^a,b^	17 ± 2 ^b,c^	48 ± 3 ^a^	633 ± 27 ^a^	316 ± 4 ^c^
*O. europaea*	187 ± 5 ^b^	2.3 ± 0.3 ^b^	28 ± 3 ^a,b^	25 ± 3 ^b,c,d^	618 ± 9 ^a^	373 ± 6 ^d^
Gallic acid	102 ± 4 ^4^	n.d.	n.d.	n.d.	n.d.	n.d.
Epicatechin	167 ± 6 ^4^	9 ± 1 ^c^	n.d.	n.d.	n.d.	n.d.
Physostigmine	n.d.	n.d.	n.d.	n.d.	n.d.	0.043 ± 0.004 ^6^
1,10-phenanthroline	n.d.	n.d.	n.d.	n.d.	83 ± 2 ^5^	n.d.
Elastatinal (51.26 ppm)	n.d.	n.d.	n.d.	53 ± 5	n.d.	n.d.
Kojic acid (21.3 ppm)	n.d.	n.d.	49 ± 6	n.d.	n.d.	n.d.

Data are means ± standard deviation (*n* = 3). ^1^ Inhibitory Concentration at 50%. ^2^ At 500 mg/L. ^3^ At 1000 mg/L. ^4^ Inhibitory Concentration at 20%. ^5^ % Inhibition. ^6^ Inhibitory Concentration at 90%. Different letters represent statistically significant differences at *p* < 0.05 level. n.d.: no data.

## Data Availability

The data presented in this study are available on request from the corresponding author.

## Supplementary Materials

The following supporting information can be downloaded at https://www.mdpi.com/article/10.3390/antiox13020217/s1, Figure S1. Base peak chromatogram from the extracts. A. *T. cacao*. B. *H. sabdariffa*. C. *S. marianum.* D. *L. citriodora*. E. *O. europaea*; Table S1. Identification of phytochemical compounds in *T. cacao* extract by HPLC-ESI-qTOF-MS; Table S2. Identification of phytochemical compounds in *H. sabdariffa* extract by HPLC-ESI-qTOF-MS; Table S3. Identification of phytochemical compounds in *S. marianum* extract by HPLC-ESI-qTOF-MS; Table S4. Identification of phytochemical compounds in *L. citriodora* extract by HPLC-ESI-qTOF-MS; Table S5. Identification of phytochemical compounds in *O. europaea* extract by HPLC-ESI-qTOF-MS; Table S6. Numerical results (mean and SD) and statistical significance for Figure 1 results; Table S7. Equivalent DMSO concentrations for each extract concentration.
