# Supplementary material for: Evaluation of Bioactive Effects of Five Plant Extracts with Different Phenolic Compositions against Different Therapeutic Targets
Source: Antioxidants (Basel). 2024 Feb 8;13(2):217. doi: 10.3390/antiox13020217 (PMC10886104; doi:10.3390/antiox13020217)
Supplement: Supplementary file 1 [file antioxidants-13-00217-s001.zip › antioxidants-2857054-supplementary.pdf]

Supplementary Material

Evaluation of bioactive effects of five plant extracts with different phenolic composition against different therapeutic targets

María del Carmen Villegas-Aguilar[1], Noelia Sánchez-Marzo[2], Álvaro Fernández-Ochoa[1], Carmen Del Río[3,4], Joan Montaner[3,4], Vicente Micol[2,5], María Herranz-López[2], Enrique Barrajón-Catalán[2], David Arráez-Román[1], María de la Luz Cádiz-Gurrea[1,*], Antonio Segura-Carretero[1]

[1] Department of Analytical Chemistry, University of Granada, 18071 Granada, Spain

[2] Institute of Research, Development and Innovation in Biotechnology of Elche (IDiBE) and Molecular and Cell Biology Institute (IBMC), Miguel Hernández University (UMH), 03202 Elche, Spain

[3] Institute of Biomedicine of Seville (IBiS), Hospital Universitario Virgen del Rocío, CSIC, Universidad de Sevilla, 41013 Seville, Spain

[4] Department of Neurology, Hospital Universitario Virgen Macarena, 41009 Seville, Spain

[5] CIBEROBN (Physiopathology of Obesity and Nutrition CB12/03/30038) Carlos III Health Institute, 28029 Madrid, Spain

Corresponding authors

* mluzcadiz@ugr.es

Figure S1. Base peak chromatogram from the extracts. A. *T. cacao*. B. *H. sabdariffa*. C. *S. marianum.* D. *L. citriodora*. E. *O. europaea*.

A

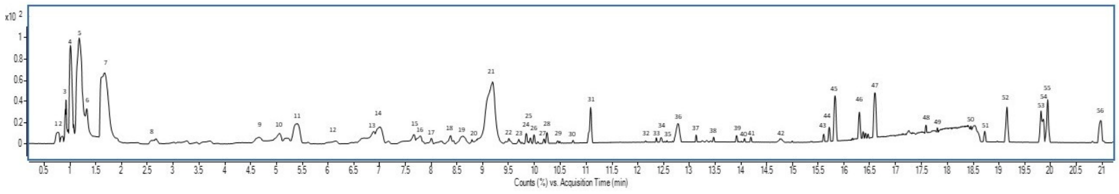

B

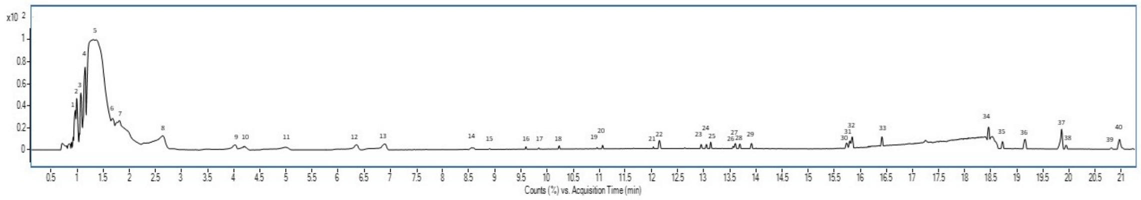

C

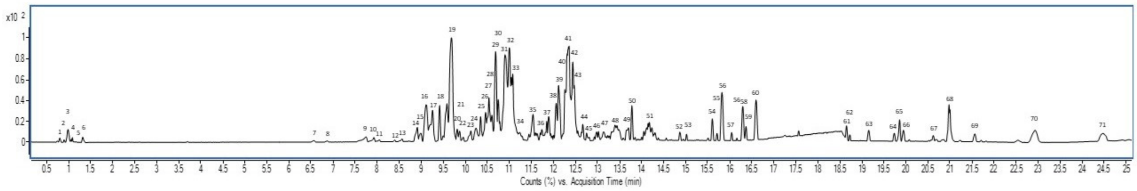

D

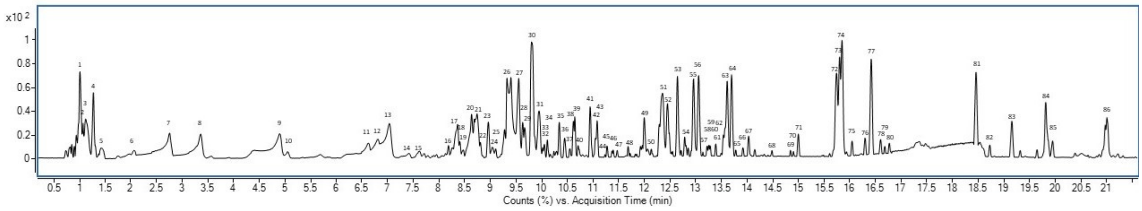

E

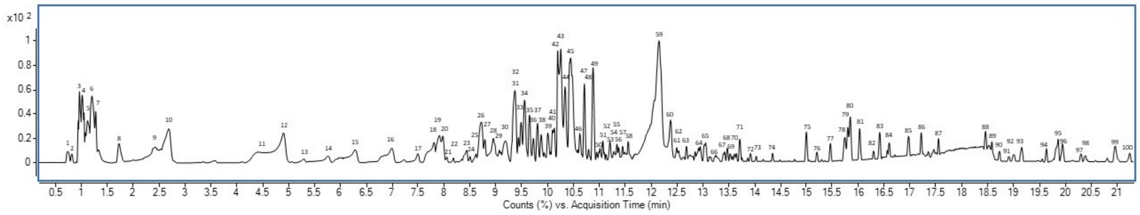

Table S1. Identification of phytochemical compounds in *T. cacao* extract by HPLC-ESI-qTOF-MS.

Peak	RT (min)	Observed [M-H]-	Theoretical [M-H]-	Mass error (ppm)	Mol. Formula	Level of annotation	Compounds	MS/MS fragments	References
1	0.75	272.9512	-	-	-	4	Unknown	-	-
2	0.91	203.1035	203.1037	-0.98	$C_8H_{16}N_2O_4$	2	Valylserine	74/141/173	Pubchem: 18218236
3	0.92	217.1187	217.1194	-3.22	$C_9H_{18}N_2O_4$	2	Leucylserine	74/129/173	Pubchem: 3621685
4	1.01	195.0519	195.0510	4.61	$C_6H_{12}O_7$	2	Gluconic acid	75/129/177	HMDB0000625
5	1.13	191.0207	191.0197	5.24	$C_6H_8O_7$	2	Citric acid isomer 1	87/111	HMDB0000094
6	1.33	191.0228	191.0197	16.23	$C_6H_8O_7$	2	Citric acid isomer 2	87/111	HMDB0000094
7	1.69	96.9605	96.9601	4.13	H_2O_4S	3	Sulfate	-	HMDB0001448
8	2.67	117.0554	117.0557	-2.56	$C_5H_{10}O_3$	2	3-Hydroxyvaleric acid	71/99	HMDB0000531
9	4.66	294.0606	294.0619	-4.42	$C_{13}H_{13}NO_7$	2	N-caffeoyl-L-aspartate isomer 1	88/132/179	(M. L. Cádiz-Gurrea et al., 2014)
10	5.05	294.0610	294.0619	-3.06	$C_{13}H_{13}NO_7$	2	N-caffeoyl-L-aspartate isomer 2	88/132/179	(M. L. Cádiz-Gurrea et al., 2014)
11	5.40	131.0704	131.0714	-7.63	$C_6H_{12}O_3$	2	L-Leucate	85	Pubchem: 69955
12	5.61	407.1554	407.1559	-1.23	$C_{17}H_{28}O_{11}$	3	Secoiridoid derived	99/263/305	(M. de la L. Cádiz-Gurrea et al., 2020)
13	6.88	278.0670	278.0670	0.00	$C_{13}H_{13}NO_6$	2	L-Aspartic acid. N-[3-(4-hydroxyphenyl)-1-oxo-2-propenyl]	71/93/119	(M. L. Cádiz-Gurrea et al., 2014)
14	7.01	165.0554	165.0557	-1.82	$C_9H_{10}O_3$	2	Dihydro-p-coumaric acid	72/103/147	HMDB0002199
15	7.60	289.0716	289.0718	-0.69	$C_{15}H_{14}O_6$	1	Catechin	161/163/174/192	HMDB0002780

16	7.80	275.1006	-	-	-	4	Unknown	-	-
17	8.01	577.1352	577.1352	0.00	$C_{30}H_{26}O_{12}$	1	Procyanidin B1	-	(M. L. Cádiz-Gurrea et al., 2014)
18	8.37	437.2040	437.2028	2.74	$C_{19}H_{34}O_{11}$	2	Ebracteatoside D isomer 1	57/99/161	-
19	8.61	167.0366	167.0350	9.58	$C_8H_8O_4$	3	Vanillic acid	-	HMDB0000484
20	8.79	865.1988	865.1985	0.35	$C_{45}H_{38}O_{18}$	2	Procyanidin C	289/577	(M. L. Cádiz-Gurrea et al., 2014)
21	9.18	305.0690	305.0667	7.54	$C_{15}H_{14}O_7$	1	Epigallocatechin	225/67/59	(Zhang et al., 2018)
22	9.51	437.2057	437.2028	6.63	$C_{19}H_{34}O_{11}$	2	Ebracteatoside D isomer 2	57/99/161	-
23	9.70	737.1750	737.1723	3.66	$C_{36}H_{34}O_{17}$	2	(Epi)catechin dimer hexose	449/611/539/289	(M. L. Cádiz-Gurrea et al., 2014)
24	9.85	463.0878	463.0882	-0.86	$C_{21}H_{20}O_{12}$	1	Quercetin glucoside	149/242/271/285/300/335	HMDB0037362
25	9.93	707.1787	707.1828	-5.80	$C_{32}H_{36}O_{18}$	2	Kalambroside A	449/539/581/289	Pubchem: 10349838
26	9.99	516.2476	516.2450	5.04	$C_{24}H_{39}NO_{11}$	4	Unknown	-	(M. L. Cádiz-Gurrea et al., 20) 14
27	10.18	326.1075	326.1034	12.57	$C_{18}H_{17}NO_5$	2	Deoxyclovamide (N-[(2E)-3-(3.4-Dihydroxyphenyl)-1-oxo-2-propen-1-yl]-L-tyrosine)	206/282/119	(M. L. Cádiz-Gurrea et al., 2014)
28	10.24	433.0806	433.0776	6.93	$C_{20}H_{18}O_{11}$	2	Quercetin arabinoside	300/271	(M. L. Cádiz-Gurrea et al., 2014)
29	10.45	238.1091	238.1085	2.52	$C_{12}H_{17}NO_4$	3	Dihydroisoflavipucine	-	Pubchem: 38354263
30	10.75	272.0935	272.0928	2.57	$C_{15}H_{15}NO_4$	2	L-Thyronine	94/124	HMDB0000667
31	11.09	421.2064	421.2079	-3.56	$C_{19}H_{34}O_{10}$	2	1-Octen-3-yl primeveroside	57/97/277	HMDB0032960

32	12.16	301.0339	301.0354	-4.98	$C_{15}H_{10}O_7$	1	Quercetin	151/121/65	HMDB0005794
33	12.36	391.1196	391.1187	2.30	$C_{23}H_{20}O_6$	2	Dehydrodeguelin	117/62	(Bini et al., 2023)
34	12.46	329.2329	329.2333	-1.21	$C_{18}H_{34}O_5$	2	5,8,12-Trihydroxy-9-octadecenoic acid isomer 1	211	HMDB0030936
35	12.57	329.2358	329.2333	7.59	$C_{18}H_{34}O_5$	2	5,8,12-Trihydroxy-9-octadecenoic acid isomer 2	211	HMDB0030936
36	12.79	357.1215	357.1191	6.72	$C_{16}H_{22}O_9$	2	Sweroside	97/198	(M. L. Cádiz-Gurrea et al., 2014)
37	13.13	394.2954	394.2963	-2.28	$C_{23}H_{41}NO_4$	4	Unknown	-	-
38	13.47	276.1227	276.1241	-5.07	$C_{15}H_{19}NO_4$	3	Barmumycin	-	(Lorente et al., 2010)
39	13.92	293.1748	293.1758	-3.41	$C_{17}H_{26}O_4$	2	Gingerol	236/221	HMDB0005783
40	14.07	333.1815	333.1820	-1.50	$C_{18}H_{26}N_2O_4$	3	Hydroxyhuperzine	-	(Jiang et al., 2010)
41	14.20	367.1671	367.1663	2.18	$C_{21}H_{24}N_2O_4$	3	3,4-dimethoxy-N-[4-[oxo(1-piperidinyl)methyl]phenyl]benzamide	-	CHEBI:114263
42	14.77	195.0686	195.0663	11.79	$C_{10}H_{12}O_4$	2	Ethyl vanillate	123	(Schwarz et al., 2021)
43	15.61	564.3295	-	-	-	4	Unknown	-	-
44	15.71	476.2780	476.2780	0.00	$C_{27}H_{41}O_7$	2	Sinapoyloxypalmitate	279	Pubchem: 90657145
45	15.83	564.3292	-	-	-	4	Unknown	-	-
46	16.30	540.3288	-	-	-	4	Unknown	-	-
47	16.60	566.3492	-	-	-	4	Unknown	-	-
48	17.58	299.2585	299.2592	-2.34	$C_{18}H_{36}O_3$	3	Hydroxyoctadecanoic acid	-	HMDB0112182
49	17.81	297.2424	297.2435	-3.70	$C_{18}H_{34}O_3$	2	Oxooctadecanoic acid	253/155	HMDB0030981

50	18.46	277.2159	277.2173	-5.05	$C_{18}H_{30}O_2$	2	Linolenic acid	205/97	HMDB0001388
51	18.73	375.2712	375.2752	-10.66	$C_{20}H_{40}O_6$	2	Myristyl glucoside	291/311	Pubchem: 6453025
52	19.16	279.2328	279.2330	-0.72	$C_{18}H_{32}O_2$	3	Linoleic acid	-	HMDB0000673
53	19.82	255.2325	255.2330	-1.96	$C_{16}H_{32}O_2$	2	Palmitic acid	190/110	HMDB0000220
54	19.87	403.3052	403.3065	-3.22	$C_{22}H_{44}O_6$	3	Hexadecyl D-glucoside	-	Pubchem: 171356
55	19.95	281.2482	281.2486	-1.42	$C_{18}H_{34}O_2$	2	Oleic Acid	211/101	HMDB0000207
56	20.98	383.1934	383.1923	2.87	$C_{16}H_{32}O_{10}$	3	Hexanedioic acid derivative	-	Pubchem: 88032455

RT: Retention Time; Mol. Formula: Molecular Formula

Table S2. Identification of phytochemical compounds in *H. sabdariffa* extract by HPLC-ESI-qTOF-MS.

Peak	RT (min)	Observed [M-H]-	Theoretical [M-H]-	Mass error (ppm)	Mol. Formula	Level of annotation	Compounds	MS/MS fragments	References
1	0.96	132.0302	132.0302	0.00	$C_4H_7NO_4$	2	Aspartic Acid	88/71	HMDB0000191
2	0.99	294.0807	294.0831	-8.16	$C_{10}H_{17}NO_9$	2	Fructose-aspartic acid	132/88	(Luo et al., 2020)
3	1.07	207.0150	207.0146	1.93	$C_6H_8O_8$	2	Hibiscus acid isomer 1	189/127	HMDB0031159
4	1.15	207.0159	207.0146	6.28	$C_6H_8O_8$	2	Hibiscus acid isomer 2	189/127	HMDB0031159
5	1.32	189.0078	189.0041	19.58	$C_6H_6O_7$	2	Hibiscus acid lactone	127	(Rodríguez-Medina et al., 2009)
6	1.66	203.0215	203.0197	8.87	$C_7H_8O_7$	2	Hibiscus acid monomethyl ester isomer 1	142/157/201	(Hifnawy et al., 2020)
7	1.82	203.0215	203.0197	8.87	$C_7H_8O_7$	2	Hibiscus acid monomethyl ester isomer 2	142/157/201	(Hifnawy et al., 2020)
8	2.63	217.0332	217.0354	-10.14	$C_8H_{10}O_7$	2	Hibiscus acid dimethylester	155/125	(Villegas-Aguilar et al., 2020)
9	4.02	353.0867	353.0878	-3.12	$C_{16}H_{18}O_9$	2	Chlorogenic acid isomer 1	191/135	HMDB0003164
10	4.20	297.0266	297.0252	4.71	$C_{12}H_{10}O_9$	2	Methyl gallate derivative isomer 1	125/82	(Pierson et al., 2014)
11	4.98	297.0266	297.0252	4.71	$C_{12}H_{10}O_9$	2	Methyl gallate derivative isomer 2	125/82	(Pierson et al., 2014)
12	6.34	353.0867	353.0878	-3.12	$C_{16}H_{18}O_9$	2	Chlorogenic acid isomer 2	191/135	HMDB0003164
13	6.89	353.0867	353.0878	-3.12	$C_{16}H_{18}O_9$	2	Chlorogenic acid isomer 3	191/135	HMDB0003164
14	8.56	335.0771	335.0772	-0.30	$C_{16}H_{16}O_8$	2	5-O-Caffeoylshikimic acid	161/191/133	(Villegas-Aguilar et al., 2020)

15	8.90	367.1030	367.1035	-1.36	$C_{17}H_{20}O_9$	2	3-O-Feruloylquinic acid	135/179	HMDB0030669
16	9.60	609.1458	609.1461	-0.49	$C_{27}H_{30}O_{16}$	3	Quercetin 3-O-rutinoside	-	HMDB0037934
17	9.84	463.0884	463.0882	0.43	$C_{21}H_{20}O_{12}$	2	Quercetin 7-glucoside	301/463	HMDB0302151
18	10.23	539.1751	539.1770	-3.52	$C_{25}H_{32}O_{13}$	3	Oleuropein	-	HMDB0035872
19	10.96	317.0294	317.0303	-2.84	$C_{15}H_{10}O_8$	2	Myrecetin	151/107	HMDB0002755
20	11.07	312.1239	312.1241	-0.64	$C_{18}H_{19}NO_4$	2	N-Feruloyltyramine	148/190	Pubchem: 6440659
21	12.04	522.3276	522.3283	-1.34	$C_{25}H_{49}NO_{10}$	4	Unknown	-	-
22	12.15	301.0339	301.0354	-4.98	$C_{15}H_{10}O_7$	1	Quercetin	151/121/65	HMDB0005794
23	12.95	329.0654	329.0667	-3.95	$C_{17}H_{14}O_7$	2	3,7-Dimethylquercetin	285/299/314	HMDB0029263
24	13.05	299.0538	299.0561	-7.69	$C_{16}H_{12}O_6$	3	Kaempferide	-	Pubchem: 5281666
25	13.13	394.2945	394.2936	2.28	$C_{20}H_{43}O_7$	4	Unknown	-	-
26	13.56	343.0821	343.0823	-0.58	$C_{18}H_{16}O_7$	3	Eupatorin isomer 1	-	HMDB0252128
27	13.61	343.0818	343.0823	-1.46	$C_{18}H_{16}O_7$	3	Eupatorin isomer 2	-	HMDB0252128
28	13.69	313.0684	313.0718	-10.86	$C_{17}H_{14}O_6$	3	Cirsimaritin	-	Pubchem: 188323
29	13.92	293.1767	293.1758	3.07	$C_{17}H_{26}O_4$	3	Gingerol	236/221	HMDB0005783
30	15.74	293.2107	293.2122	-5.12	$C_{18}H_{30}O_3$	3	Hydroxylinolenic acid isomer 1	-	HMDB0011108
31	15.81	293.2119	293.2122	-1.02	$C_{18}H_{30}O_3$	3	Hydroxylinolenic acid isomer 2	-	HMDB0011108
32	15.85	293.2113	293.2122	-3.07	$C_{18}H_{30}O_3$	3	Hydroxylinolenic acid isomer 3	-	HMDB0011108
33	16.41	295.2265	295.2279	-4.74	$C_{18}H_{32}O_3$	3	Hydroxylinoleic acid	-	HMDB0247599
34	18.46	277.2159	277.2173	-5.05	$C_{18}H_{30}O_2$	2	Linolenic acid	205/97	HMDB0001388

35	18.73	375.2712	375.2752	-10.66	$C_{20}H_{40}O_6$	2	Myristyl glucoside	291/311	Pubchem: 6453025
36	19.15	279.2328	279.2330	-0.72	$C_{18}H_{32}O_2$	3	Linoleic acid	-	HMDB0000673
37	19.86	403.3052	403.3065	-3.22	$C_{22}H_{44}O_6$	3	Hexadecyl D-glucoside	-	Pubchem: 171356
38	19.94	281.2508	281.2486	7.82	$C_{18}H_{34}O_2$	2	Oleic Acid	211/101	HMDB0000207
39	20.81	443.2490	443.2439	11.51	$C_{26}H_{36}O_6$	3	Bufotalin	-	Pubchem: 12302120
40	20.97	383.1916	383.1923	-1.83	$C_{16}H_{32}O_{10}$	3	Hexanedioic acid derivative	-	Pubchem: 88032455

RT: Retention Time; Mol. Formula: Molecular Formula

Table S3. Identification of phytochemical compounds in *S. marianum* extract by HPLC-ESI-qTOF-MS.

	RT (min)	Observed [M-H]-	Theoretical [M-H]-	Mass error (ppm)	Mol. Formula	Level of annotation	Compounds	MS/MS fragments	References
1	0.80	112.9866	112.9880	-12.39	$C_4H_2O_4$	3	Acetylenedicarboxylic acid	-	HMDB0247933
2	0.90	134.0479	134.0472	5.22	$C_5H_5N_5$	3	Adenine	-	HMDB0000034
3	0.99	179.0556	179.0561	-2.79	$C_6H_{12}O_6$	3	Fructose	-	HMDB0000660
4	1.06	266.0883	266.0894	-4.13	$C_{10}H_{13}N_5O_4$	3	Adenosine	-	HMDB0000050
5	1.09	117.0130	-	-	-	4	Unknown	-	-
6	1.32	197.8088	-	-	-	4	Unknown	-	-
7	6.57	303.0492	303.0510	-5.94	$C_{15}H_{12}O_7$	2	Dihydroquercetin	285/125	(Ferysiuk et al., 2020)
8	6.87	353.0881	353.0878	0.85	$C_{16}H_{18}O_9$	2	Chlorogenic acid	191/161	HMDB0003164
9	7.75	373.1487	373.1504	-4.56	$C_{17}H_{26}O_9$	2	Deoxyloganin	165/135	Pubchem: 440906
10	7.93	441.1949	441.1978	-6.57	$C_{18}H_{34}O_{12}$	2	Hexo-Glucose	395/249	Pubchem: 129630443
11	8.05	505.1915	505.1927	-2.38	$C_{22}H_{34}O_{13}$	2	Oleuropeic acid derivative isomer 1	459/293	(Tian et al., 2009)
12	8.41	475.1843	475.1821	4.63	$C_{21}H_{32}O_{12}$	3	Cistanoside E	-	Pubchem: 21632979
13	8.58	505.1935	505.1927	1.58	$C_{22}H_{34}O_{13}$	2	Oleuropeic acid derivative isomer 2	459/293	(Tian et al., 2009)
14	8.92	459.1856	459.1872	-3.48	$C_{21}H_{32}O_{11}$	2	Apiosylepirhododendrin	89/59	Pubchem: 101287073
15	8.99	163.0389	163.0401	-7.36	$C_9H_8O_3$	2	4-Hydroxycinnamic acid	119	HMDB0002035
16	9.12	193.0141	193.0142	-0.52	$C_9H_6O_5$	2	Trihydroxycoumarin	137/93	(Waseem et al., 2021)
17	9.26	481.1161	481.1140	4.36	$C_{25}H_{22}O_{10}$	2	Silybin isomer 1	125/178	MoNA:VF-NPL-QTOF009680

18	9.42	455.2130	455.2134	-0.88	$C_{19}H_{36}O_{12}$	3	Alkyl glicoside	-	(Vidal & Ccapatinta, 2018)
19	9.58	303.0512	303.0510	0.66	$C_{15}H_{12}O_7$	2	Dihydroquercetin	285/125	(Ferysiuk et al., 2020)
20	9.73	607.1081	607.1093	-1.98	$C_{30}H_{24}O_{14}$	2	(Epi)gallocatechin-A-(epi)gallocatechin	285/303	(Singh et al., 2018)
21	9.82	187.0957	187.0976	-10.16	$C_9H_{16}O_4$	3	Azelaic acid	-	HMDB0000784
22	9.87	433.1123	433.1140	-3.93	$C_{21}H_{22}O_{10}$	2	Naringenin 4'-O-glucoside	271/151	Pubchem: 42607906
23	10.12	481.1125	481.1140	-3.12	$C_{25}H_{22}O_{10}$	2	Silybin isomer 2	125/178	MoNA:VF-NPL-QTOF009680
24	10.25	483.1293	483.1297	-0.83	$C_{25}H_{24}O_{10}$	2	Silybin hydrogenated	125/151/285	(Vrba et al., 2018)
25	10.35	813.3162	813.3187	-3.07	$C_{38}H_{54}O_{19}$	2	Tricrocin	767/473	Pubchem: 22833598
26	10.46	383.1604	383.1612	-2.09	$C_{21}H_{24}N_2O_5$	3	Alschomine	-	Pubchem: 11969856
27	10.54	675.3212	675.3174	5.63	$C_{39}H_{48}O_{10}$	2	Gambogoic acid A	629/293	ChemBK: 1592842-93-7
28	10.61	481.1120	481.1140	-4.16	$C_{25}H_{22}O_{10}$	2	Silybin isomer 3	125/178	MoNA:VF-NPL-QTOF009680
29	10.69	287.0516	287.0561	-15.68	$C_{15}H_{12}O_6$	2	Aromadendrin	125/259	HMDB0030847
30	10.75	317.0655	317.0667	-3.78	$C_{16}H_{14}O_7$	2	(+)-Dihydroisorhamnetin	245/259	HMDB0037501
31	10.96	481.1114	481.1140	-5.40	$C_{25}H_{22}O_{10}$	2	Silychrystin isomer 1	151/125	MoNA:RIKENPlaSMA007844
32	11.00	481.1139	481.1140	-0.21	$C_{25}H_{22}O_{10}$	2	Silybin isomer 3	125/178	MoNA:VF-NPL-QTOF009680
33	11.03	481.1150	481.1140	2.08	$C_{25}H_{22}O_{10}$	2	Silychrystin isomer 2	107/125/151/325/463	MoNA:RIKENPlaSMA007844
34	11.08	481.1146	481.1140	1.25	$C_{25}H_{22}O_{10}$	2	Silychrystin isomer 3	125/151/178/325/355/433	MoNA:MetaboBASE0687
35	11.54	677.1892	677.1876	2.36	$C_{35}H_{34}O_{14}$	4	Unknown	-	-

36	11.85	685.3060	685.3077	-2.48	$C_{33}H_{50}O_{15}$	2	Pterocecide B	477/639	Pubchem: 122228272
37	11.90	659.1765	659.1770	-0.76	$C_{35}H_{32}O_{13}$	2	Phylloflavanine	178/125	(Foo, 1987)
38	12.06	481.1130	481.1140	-2.08	$C_{25}H_{22}O_{10}$	2	Silybin isomer 4	125/152/301	MoNA:VF-NPL-QTOF009680
39	12.12	481.1176	481.1140	7.48	$C_{25}H_{22}O_{10}$	2	Silybin isomer 5	125/152/301	MoNA:VF-NPL-QTOF009680
40	12.26	481.1172	481.1140	6.65	$C_{25}H_{22}O_{10}$	2	Silybin isomer 6	125/152/273	MoNA:VF-NPL-QTOF009680
41	12.34	481.1158	481.1140	3.74	$C_{25}H_{22}O_{10}$	2	Silybin isomer 7	125/152/301	MoNA:VF-NPL-QTOF009680
42	12.44	481.1120	481.1140	-4.16	$C_{25}H_{22}O_{10}$	2	Silybin isomer 8	125/152/301	MoNA:VF-NPL-QTOF009680
43	12.48	481.1156	481.1140	3.33	$C_{25}H_{22}O_{10}$	2	Silybin isomer 9	125/152/301	MoNA:VF-NPL-QTOF009680
44	12.67	381.2269	381.2282	-3.41	$C_{21}H_{34}O_6$	3	Sarcostin	-	Pubchem: 46173994
45	12.94	477.0837	477.0827	2.10	$C_{25}H_{18}O_{10}$	3	Pradinone I	-	Pubchem: 460846
46	13.01	477.2489	477.2494	-1.05	$C_{26}H_{38}O_8$	3	Adenanthin B isomer 1	-	(Hu et al., 2019)
47	13.12	811.1896	811.1938	-5.18	$C_{35}H_{40}O_{22}$	3	Kaempferol 3-[2''-glucosyl-6''-acetylgalactoside] 7-glucoside	-	FDB016214
48	13.42	989.2502	989.2510	-0.81	$C_{52}H_{46}O_{20}$	4	Unknown	-	-
49	13.70	523.1249	523.1246	0.57	$C_{27}H_{24}O_{11}$	3	Acetylsilybin A/B	-	Pubchem: 10236891
50	13.78	477.2522	477.2493	6.08	$C_{26}H_{38}O_8$	3	Adenanthin B isomer 2	-	(Hu et al., 2019)
51	14.14	479.0975	479.0984	-1.88	$C_{25}H_{20}O_{10}$	2	Dehydrosilybin	125/177/179/283/327/449	HMDB0040513
52	14.87	627.1569	627.1567	0.32	$C_{27}H_{32}O_{17}$	2	Vitexia-glucoside	152/271/435/463	Pubchem: 56933064
53	15.02	313.2378	313.2384	-1.92	$C_{18}H_{34}O_4$	3	Octadecanedioic acid	-	Pubchem: 70095
54	15.61	564.3351	-	-	-	4	Unknown	-	-
55	15.71	476.2772	476.2780	-1.68	$C_{27}H_{41}O_7$	3	Sinapoyloxypalmitate	-	Pubchem: 90657145

56	15.83	564.3364	-	-	-	4	Unknown	-	-
57	16.04	540.3289	540.3304	-2.78	$C_{29}H_{49}O_9$	3	Steroid compound	-	(Hendriani & Yulinah Sukandar, 2016)
58	16.30	540.3306	540.3304	0.37	$C_{29}H_{49}O_9$	3	Steroid compound	-	(Hendriani & Yulinah Sukandar, 2016)
59	16.37	566.3453	-	-	-	4	Unknown	-	-
60	16.60	566.3530	-	-	-	4	Unknown	-	-
61	18.66	591.4117	591.4114	0.51	$C_{31}H_{60}O_{10}$	4	Unknown	-	-
62	18.73	375.2712	375.2752	-10.66	$C_{20}H_{40}O_6$	2	Myristyl glucoside	291/311	Pubchem: 6453025
63	19.16	279.2328	279.2330	-0.72	$C_{18}H_{32}O_2$	3	Linoleic acid	-	HMDB0000673
64	19.74	577.3730	577.3746	-2.77	$C_{33}H_{54}O_8$	3	Timosaponin A	-	(Wang et al., 2021)
65	19.85	403.3052	403.3065	-3.22	$C_{22}H_{44}O_6$	3	Hexadecyl D-glucoside	-	Pubchem: 171356
66	19.94	281.2508	281.2486	7.82	$C_{18}H_{34}O_2$	2	Oleic Acid	211/101	HMDB0000207
67	20.62	605.4040	605.4059	-3.14	$C_{35}H_{58}O_8$	3	Deoxybafilomycin A1	-	CAS: 1883587-79-8
68	20.98	621.4417	621.4372	7.24	$C_{36}H_{62}O_8$	3	Ginsenoside Rh2	-	Pubchem:119307
69	21.56	633.4375	633.4371	0.63	$C_{37}H_{62}O_8$	3	Karaviloside III	-	CHEMBL2335924
70	22.92	371.2592	371.2592	0.00	$C_{24}H_{36}O_3$	3	Pelandjauic acid	-	Pubchem: 178575
71	24.49	373.2751	373.2748	0.80	$C_{24}H_{38}O_3$	3	Dehydrolithocholic acid	-	Pubchem: 4446994

RT: Retention Time; Mol. Formula: Molecular Formula

Table S4. Identification of phytochemical compounds in *L. citriodora* extract by HPLC-ESI-qTOF-MS.

Peak	Rt (min)	Observed [M-H]-	Theoretical [M-H]-	Mass error (ppm)	Mol. Formula	Level of annotation	Compounds	MS/MS fragments	References
1	1.01	195.0511	195.0510	0.51	$C_6H_{12}O_7$	2	Gluconic acid	75/105/177	HMDB0184581
2	1.07	133.014	133.0142	-1.50	$C_4H_6O_5$	3	Malic acid isomer 1	-	CAS: 6915-15-7
3	1.14	133.0141	133.0142	-0.75	$C_4H_6O_6$	3	Malic acid isomer 2	-	CAS: 6915-15-7
4	1.27	317.0452	317.0514	-19.56	$C_{12}H_{14}O_{10}$	4	Unknown	-	-
5	1.47	303.8787	-	-	-	4	Unknown	-	-
6	2.07	391.1232	391.1246	-3.58	$C_{16}H_{24}O_{11}$	2	Shanziside	229/185/167/149/123	(Quirantes-Piné et al., 2010)
7	2.73	373.1101	373.114	-10.45	$C_{16}H_{22}O_{10}$	1	Gardoside	123/149/211	(Quirantes-Piné et al., 2010)
8	3.35	461.1642	461.1665	-4.99	$C_{20}H_{30}O_{12}$	2	Verbasoside	135/315	(Quirantes-Piné et al., 2010)
9	4.90	389.1091	389.1089	0.51	$C_{16}H_{22}O_{11}$	2	Theveside	121/69	(Quirantes-Piné et al., 2009)
10	5.06	487.1446	487.1457	-2.26	$C_{21}H_{28}O_{13}$	3	Cistanoside F	-	(Quirantes-Piné et al., 2009)
11	6.61	387.1617	387.1661	-11.36	$C_{18}H_{28}O_9$	2	Tuberonic acid glucoside	59/89	Pubchem: 5281204
12	6.81	431.1916	431.1923	-1.62	$C_{20}H_{32}O_{10}$	3	Sacranoside A	-	Pubchem: 102094959
13	7.01	433.2064	433.2079	-3.46	$C_{20}H_{34}O_{10}$	2	Monoterpene derivative	387/89	(Li et al., 2019)

14	7.41	225.1136	225.1132	1.78	$C_{12}H_{12}O_4$	2	Eugenitin	59	Pubchem: 3083581
15	7.63	307.1405	307.1398	2.28	$C_{13}H_{24}O_8$	4	Unknown	-	-
16	8.19	593.1497	593.1512	-2.53	$C_{27}H_{30}O_{15}$	3	Kaempferol 3-O-rutinoside	-	HMDB0302426
17	8.25	641.2095	641.2087	1.25	$C_{29}H_{38}O_{16}$	3	Yadanzioside I	-	Pubchem: 10484290
18	8.37	435.2226	435.2236	-2.30	$C_{20}H_{36}O_{10}$	3	Deniose	-	Pubchem: 10741574
19	8.48	377.1814	377.1817	-0.80	$C_{17}H_{30}O_9$	4	Unknown	-	-
20	8.64	639.1863	639.1931	-10.64	$C_{29}H_{36}O_{16}$	3	Plantamajoside	-	Pubchem: 5281788
21	8.75	377.1814	377.1817	-0.80	$C_{17}H_{30}O_9$	4	Unknown	-	-
22	8.80	553.1575	553.1563	2.17	$C_{25}H_{30}O_{14}$	2	Lippioside II	161/135	131751542
23	8.96	353.1822	353.1817	1.42	$C_{15}H_{30}O_9$	3	Hydroxy-PEG6-acid	-	Pubchem: 60146229
24	9.28	653.2077	653.2087	-1.53	$C_{30}H_{38}O_{16}$	2	Campneoside I	621	(Quirantes-Piné et al., 2009)
25	9.33	623.2	623.1981	3.05	$C_{29}H_{36}O_{15}$	1	Verbascoside isomer 1	153/161/179/487	HMDB0034843
26	9.40	623.1913	623.1981	-10.91	$C_{29}H_{36}O_{15}$	1	Verbascoside isomer 2	153/161/179/487	HMDB0034843
27	9.56	623.1921	623.1981	-9.63	$C_{29}H_{36}O_{15}$	1	Verbascoside isomer 3	153/161/179/487	HMDB0034843
28	9.64	417.2128	417.213	-0.48	$C_{20}H_{34}O_9$	3	Maryal	-	Pubchem: 5320270
29	9.67	651.1231	-	-	-	4	Unknown	-	-
30	9.82	623.2007	623.1981	4.17	$C_{29}H_{36}O_{15}$	1	Verbascoside isomer 3	153/161/179/487	HMDB0034843

31	9.96	637.2131	637.2138	-1.10	$C_{30}H_{38}O_{15}$	3	Verbaspinoside isomer 1	-	Pubchem: 23928137
32	10.02	521.1645	521.1665	-3.84	$C_{25}H_{30}O_{12}$	3	Linearoside	-	Pubchem: 102066680
33	10.05	551.1693	551.1770	-13.97	$C_{26}H_{32}O_{13}$	2	Durantoside I	175/151/235	Pubchem: 95223135
34	10.11	463.2523	463.2549	-5.61	$C_{22}H_{40}O_{10}$	3	Methyladoligose E	-	Pubchem: 101921401
35	10.34	467.2105	467.2075	6.42	$C_{27}H_{32}O_{7}$	3	Mexicanolide	-	Pubchem: 267328
36	10.45	637.2162	637.2138	3.77	$C_{30}H_{38}O_{15}$	3	Verbaspinoside isomer 2	-	23928137
37	10.55	635.1270	635.1254	2.52	$C_{28}H_{28}O_{17}$	3	Acacetin 7-diglucuronide	-	Pubchem: 11968442
38	10.62	651.2308	651.2294	2.15	$C_{31}H_{40}O_{15}$	3	Martynoside	-	HMDB0254342
39	10.64	335.1708	335.1711	-0.90	$C_{15}H_{28}O_{8}$	4	Unknown	-	-
40	10.71	595.1654	595.1668	-2.35	$C_{27}H_{32}O_{15}$	2	Neoeriocitrin	163/87	Pubchem: 114627
41	10.94	591.2074	591.2083	-1.52	$C_{29}H_{36}O_{13}$	2	Osmanthuside B	161/113	(Leyva-Jiménez et al., 2018)
42	11.04	569.2231	569.2240	-1.58	$C_{27}H_{38}O_{13}$	2	Sesinoside	161/188/359	Pubchem: 102502279
43	11.09	337.1870	337.1868	0.59	$C_{15}H_{30}O_{8}$	4	Unknown	-	-
44	11.28	681.2200	681.2189	1.61	$C_{35}H_{38}O_{14}$	2	PinoresinolO-[6-O-(E)-caffeoyl]-β-D-glucopyranoside	323/161	Pubchem: 17410
45	11.37	361.1878	361.1868	2.77	$C_{17}H_{30}O_{8}$	3	Glyceryl octyl ascorbic acid	-	Pubchem: 54588053
46	11.40	895.3534	-	-	-	4	Unknown	-	-

47	11.48	181.0875	181.0870	2.76	$C_{10}H_{14}O_3$	3	3,4-Dimethoxyphenethyl alcohol	-	Pubchem: 81911
48	11.67	579.1697	579.1719	-3.80	$C_{27}H_{32}O_{14}$	2	Naringin	163/137	HMDB0002927
49	11.99	315.0493	315.0510	-5.40	$C_{16}H_{12}O_7$	2	Isorhamnetin isomer 1	271/300	HMDB0002655
50	12.29	481.1147	481.1140	1.45	$C_{25}H_{22}O_{10}$	1	Silybin isomer 1	125/152/179/180/301	MoNA:FIO01021
51	12.34	481.1124	481.1140	-3.33	$C_{25}H_{22}O_{11}$	1	Silybin isomer 2	125/152/179/180/302	MoNA:FIO01022
52	12.44	481.1131	481.1140	-1.87	$C_{25}H_{22}O_{12}$	1	Silybin isomer 3	125/152/179/180/303	MoNA:FIO01023
53	12.64	329.0647	329.0667	-6.08	$C_{17}H_{14}O_7$	2	3,7-Dimethylquercetin isomer 1	285/299/314	HMDB0029263
54	12.79	599.2689	599.2709	-3.34	$C_{29}H_{44}O_{13}$	2	Jatamanvaltrate C	162/243/291/389	-
55	12.96	329.0668	329.0667	0.30	$C_{17}H_{14}O_7$	2	3,7-Dimethylquercetin isomer 2	285/299/314	HMDB0029263
56	13.03	299.0546	299.0561	-5.02	$C_{16}H_{12}O_6$	2	Chrysoeriol	183	HMDB0030667
57	13.14	394.2954	-	-	-	4	Unknown	-	-
58	13.22	309.2061	309.2071	-3.23	$C_{18}H_{30}O_4$	3	13(S)-Hydroperoxylinoleic acid isomer 1	-	HMDB0003871
59	13.26	315.0502	315.0510	-2.54	$C_{16}H_{12}O_7$	2	Isorhamnetin isomer 2	271/300	HMDB0002655
60	13.28	309.2058	309.2071	-4.20	$C_{18}H_{30}O_4$	3	13(S)-Hydroperoxylinoleic acid isomer 2	-	HMDB0003871
61	13.39	297.1698	297.1707	-3.03	$C_{16}H_{26}O_5$	3	Dihydroartemisinin methyl ether	-	Pubchem: 68911
62	13.53	307.1917	307.1915	0.65	$C_{18}H_{28}O_4$	2	Dihydrocapsiate	235/185/121	HMDB0034781
63	13.61	343.0813	343.0823	-2.91	$C_{18}H_{16}O_7$	2	Quercetin 3′,4′,7-trimethyl ether	313/163	Pubchem: 5748558
64	13.70	313.0697	313.0717	-6.39	$C_{17}H_{14}O_6$	2	Cirsimaritin isomer 1	313	HMDB0250276

65	13.78	373.0924	373.0929	-1.34	$C_{19}H_{18}O_8$	2	Chrysosplenetin	315/256	Pubchem: 5281608
66	13.92	293.1747	293.1758	-3.75	$C_{17}H_{26}O_4$	3	Gingerol	-	HMDB0005783
67	14.03	305.1731	305.1758	-8.85	$C_{18}H_{26}O_4$	2	9,16-Dioxo-10,12,14-octadecatrienoic acid	135/206/249	Pubchem: 18546963
68	14.49	313.0697	313.0717	-6.39	$C_{17}H_{14}O_6$	2	Cirsimaritin isomer 2	313	HMDB0250276
69	14.84	413.2567	413.2545	5.32	$C_{22}H_{38}O_7$	3	Ascorbyl palmitate isomer 1	-	HMDB0039883
70	14.90	413.2524	413.2545	-5.08	$C_{22}H_{38}O_7$	3	Ascorbyl palmitate isomer 2	-	HMDB0039883
71	14.99	721.3661	721.3652	1.25	$C_{34}H_{58}O_{16}$	4	Unknown	-	-
72	15.74	293.2107	293.2122	-5.12	$C_{18}H_{30}O_3$	3	Hydroxylinolenic acid isomer 1	-	HMDB0011108
73	15.80	293.2119	293.2122	-1.02	$C_{18}H_{30}O_3$	3	Hydroxylinolenic acid isomer 2	-	HMDB0011108
74	15.85	293.2113	293.2122	-3.07	$C_{18}H_{30}O_3$	3	Hydroxylinolenic acid isomer 3	-	HMDB0011108
75	16.05	699.3800	699.3809	-1.29	$C_{32}H_{60}O_{16}$	3	Propargyl-PEG14-acid	-	Pubchem: 102514849
76	16.30	540.3306	-	-	-	4	Unknown	-	-
77	16.42	295.2282	295.2279	1.02	$C_{18}H_{32}O_3$	3	Hydroxylinoleic acid	-	HMDB0247599
78	16.60	566.3530	566.3460	12.36	$C_{31}H_{51}O_9$	4	Unknown	-	-
79	16.68	293.2109	293.2122	-4.43	$C_{18}H_{30}O_3$	3	Hydroxylinolenic acid isomer 4	-	HMDB0011108
80	16.77	293.2120	293.2122	-0.68	$C_{18}H_{30}O_3$	3	Hydroxylinolenic acid isomer 4	-	HMDB0011108
81	18.46	277.2159	277.2173	-5.05	$C_{18}H_{30}O_2$	2	Linolenic acid	205/97	HMDB0001388
82	18.73	375.2712	375.2693	5.06	$C_{27}H_{36}O$	2	10'-Apo-beta-carotenal	116	HMDB0059605
83	19.16	279.2328	279.2330	-0.72	$C_{18}H_{32}O_2$	3	Linoleic acid	-	HMDB0000673

84	19.82	255.2325	255.2330	-1.96	$C_{16}H_{32}O_2$	2	Palmitic acid	190/110	HMDB0000220
85	19.95	281.2482	281.2486	-1.42	$C_{18}H_{34}O_2$	2	Oleic Acid	211/101	HMDB0000207
86	20.98	621.4417	621.4372	7.24	$C_{36}H_{62}O_8$	3	Ginsenoside Rh2	-	Pubchem: 119307

RT: Retention Time; Mol. Formula: Molecular Formula

Table S5. Identification of phytochemical compounds in *O. europaea* extract by HPLC-ESI-qTOF-MS.

Peak	RT (min)	Observed [M-H]-	Theoretical [M-H]-	Mass error (ppm)	Mol. Formula	Level of annotation	Compounds	MS/MS fragments	References
1	0.94	194.0424	194.0426	-1.03	$C_6H_{10}O_7$	3	Glucuronic acid	-	HMDB0000127
2	0.96	181.0721	181.0718	1.66	$C_6H_{14}O_6$	2	Sorbitol	89/101	HMDB0000247
3	1.02	195.0512	195.0510	1.03	$C_6H_{12}O_7$	2	Gluconic acid	75/105/177	HMDB0184581
4	1.05	383.1200	383.1195	1.31	$C_{14}H_{24}O_{12}$	3	Acetylsaccharose	-	Pubchem: 129641662
5	1.13	133.0143	133.0142	0.75	$C_4H_6O_5$	3	Malic acid	-	(Nergiz & Ergönül, 2009)
6	1.20	191.0545	191.0561	-8.37	$C_7H_{12}O_6$	1	Quinic acid	85/127	HMDB03072
7	1.27	317.0535	-	-	-	4	Unknown	-	-
8	1.72	96.9605	96.9601	4.13	H_2O_4S	3	Sulfate	-	HMDB0001448
9	2.42	153.0551	153.0557	-3.92	$C_8H_{10}O_3$	2	Hydroxytyrosol isomer 1	123/93	HMDB0005784
10	2.69	153.0549	153.0557	-5.23	$C_8H_{10}O_3$	2	Hydroxytyrosol isomer 2	123/93	HMDB0005784
11	4.39	389.1113	389.1089	6.17	$C_{16}H_{22}O_{11}$	2	Oleoside isomer 1	121/59/69	(Fu et al., 2023)
12	4.91	389.1078	389.1089	-2.83	$C_{16}H_{22}O_{11}$	2	Oleoside isomer 2	121/59/69	(Fu et al., 2023)
13	5.31	611.1881	611.1829	8.51	$C_{24}H_{36}O_{18}$	3	2-[2-hydroxy-3-[4-[2-hydroxy-3-(1,2,3-tricarboxypropan-2-yloxy) propoxy]cyclohexyl]oxypropoxy]propane-1,2,3-tricarboxylic acid	-	Pubchem: 59999877
14	5.75	447.1487	447.1508	-4.70	$C_{19}H_{28}O_{12}$	2	Hydroxytyrosol derivative	285	(Garcia-Aloy et al., 2020)

15	6.29	403.1208	403.1246	-9.43	$C_{17}H_{24}O_{11}$	2	Oleoside 11-methyl ester isomer 1	165/223	Pubchem: 10692563
16	7.01	377.1453	377.1453	0.00	$C_{16}H_{26}O_{10}$	2	Aldehydic form of decarboxymethyl elenolic acid glucoside	153/197	(Garcia-Aloy et al., 2020)
17	7.48	519.1702	519.1719	-3.27	$C_{22}H_{32}O_{14}$	3	Segetoside A	-	Pubchem: 102316547
18	7.81	403.1239	403.1246	-1.74	$C_{17}H_{24}O_{11}$	2	Oleoside 11-methyl ester isomer 2	165/223	Pubchem: 10692563
19	7.91	461.1678	461.1664	3.04	$C_{20}H_{30}O_{12}$	2	Verbasoside isomer 1	135/161/315	(Quirantes-Piné et al., 2010)
20	7.98	461.1657	461.1664	-1.52	$C_{20}H_{30}O_{12}$	2	Verbasoside isomer 1	135/161/315	(Quirantes-Piné et al., 2010)
21	8.05	555.1823	555.1719	18.73	$C_{25}H_{32}O_{14}$	2	Hydroxyoleuropein isomer 1	151	(Katsinas et al., 2021)
22	8.18	593.1550	593.1512	6.41	$C_{27}H_{30}O_{15}$	2	Vicenin 2	353/383/473	(Bouaziz et al., 2005)
23	8.42	401.1802	401.1817	-3.74	$C_{19}H_{30}O_{9}$	2	Methyl 7-epi-12-hydroxyjasmonate glucoside	221/59	Pubchem: 131751189
24	8.51	581.2795	581.2815	-3.44	$C_{26}H_{46}O_{14}$	4	Unknown	-	(Baker & Regg, 2018)
25	8.62	609.1487	609.1461	4.27	$C_{27}H_{30}O_{16}$	2	Quercetin 3-O-rutinoside	271/300/301	HMDB0257021
26	8.72	195.0660	195.0663	-1.54	$C_{10}H_{12}O_{4}$	2	Hydroxytyrosol Acetate	59	Pubchem: 155240
27	8.79	557.2455	557.2426	5.20	$C_{27}H_{42}O_{10}S$	3	Thiocladospolide J	-	(Salvatore et al., 2021)

28	8.96	555.1679	555.1719	-7.20	$C_{25}H_{32}O_{14}$	2	Hydroxyoleuropein isomer 2	151	(Katsinas et al., 2021)
29	9.07	525.1620	525.1614	1.14	$C_{24}H_{30}O_{13}$	2	Demethyloleuropein	61/121/165	Pubchem: 6450302
30	9.19	305.0662	305.0667	-1.64	$C_{15}H_{14}O_{7}$	2	Epigallocatechin	125	HMDB0038361
31	9.37	623.1978	623.1981	-0.48	$C_{29}H_{36}O_{15}$	1	Verbascoside isomer 1	161/315/461	(Attia et al., 2018)
32	9.43	551.2700	551.2709	-1.63	$C_{25}H_{44}O_{13}$	3	Norisoprenoid derivative	-	(Cebrián-Tarancón et al., 2021)
33	9.49	701.2317	701.2298	2.71	$C_{31}H_{42}O_{18}$	2	Oleuropein-glucoside isomer 1	139/357/481/566	Pubchem: 102031346
34	9.55	701.2300	701.2298	0.29	$C_{31}H_{42}O_{18}$	2	Oleuropein-glucoside isomer 2	139/357/481/566	Pubchem: 102031346
35	9.65	447.0934	447.0933	0.22	$C_{21}H_{20}O_{11}$	3	Luteolin 3'-glucoside	-	Pubchem: 12309350
36	9.72	701.2291	701.2298	-1.00	$C_{31}H_{42}O_{18}$	2	Oleuropein-glucoside isomer 3	139/357/481/566	Pubchem: 102031346
37	9.81	623.1947	623.1981	-5.46	$C_{29}H_{36}O_{15}$	1	Verbascoside isomer 2	161/315/461	(Attia et al., 2018)
38	9.88	607.1936	607.2032	-15.81	$C_{29}H_{36}O_{14}$	3	Isosyringalide 3'-rhamnoside	-	Pubchem: 21629997
39	10.01	539.1779	539.1770	1.67	$C_{25}H_{32}O_{13}$	1	Oleuropein isomer 1	139/205/275/307/377	HMDB0035872
40	10.10	569.1834	569.1876	-7.38	$C_{26}H_{34}O_{14}$	2	Methoxyoleuropein isomer 1	151	HMDB0035445
41	10.13	569.1876	569.1876	0.00	$C_{26}H_{34}O_{14}$	2	Methoxyoleuropein isomer 2	151	HMDB0035445
42	10.20	539.1808	539.1770	7.05	$C_{25}H_{32}O_{13}$	1	Oleuropein isomer 2	139/205/275/307/377	HMDB0035872
43	10.26	539.1811	539.1770	7.60	$C_{25}H_{32}O_{13}$	1	Oleuropein isomer 3	139/205/275/307/377	HMDB0035872

44	10.34	539.1736	539.1770	-6.31	$C_{25}H_{32}O_{13}$	1	Oleuropein isomer 4	139/205/275/307/377	HMDB0035872
45	10.46	539.1746	539.1770	-4.45	$C_{25}H_{32}O_{13}$	1	Oleuropein isomer 5	139/205/275/307/377	HMDB0035872
46	10.63	539.1781	539.1770	2.04	$C_{25}H_{32}O_{13}$	1	Oleuropein isomer 5	139/205/275/307/377	HMDB0035872
47	10.71	601.2208	601.2138	11.64	$C_{27}H_{38}O_{15}$	3	Rubricauloside	-	Pubchem: 196862
48	10.80	555.2057	555.2083	-4.68	$C_{26}H_{36}O_{13}$	3	11-Hydroxyiridodial glucoside pentaacetate	121/69/183	CSID391583
49	10.88	523.1812	523.1821	-1.72	$C_{25}H_{32}O_{12}$	2	Ligstroside	361	HMDB0034751
50	11.01	793.2908	793.2924	-2.02	$C_{38}H_{50}O_{18}$	4	Unknown	-	(Garcia-Aloy et al., 2020)
51	11.07	377.1218	377.1242	-6.36	$C_{19}H_{22}O_{8}$	2	Oleuropein aglycone isomer 1	95/139	HMDB0301749
52	11.14	553.1924	553.1927	-0.54	$C_{26}H_{34}O_{13}$	3	4''-Methyloleuropein	-	C00056345
53	11.20	377.1233	377.1242	-2.39	$C_{19}H_{22}O_{8}$	2	Oleuropein aglycone isomer 2	95/139	HMDB0301749
54	11.28	377.1208	377.1242	-9.02	$C_{19}H_{22}O_{8}$	2	Oleuropein aglycone isomer 3	95/139	HMDB0301749
55	11.34	393.1162	393.1191	-7.38	$C_{19}H_{22}O_{9}$	2	10-hydroxyoleuropein aglycone isomer 1	137/181	(Serrano-García et al., 2022)
56	11.38	377.1223	377.1242	-5.04	$C_{19}H_{22}O_{8}$	2	Oleuropein aglycone isomer 4	95/139	HMDB0301749
57	11.44	623.1403	623.1406	-0.48	$C_{31}H_{28}O_{14}$	3	Isorhamnetin-3-O-β-D-(6-p-coumaroyl) glucoside	-	(Romero-Márquez et al., 2023)
58	11.55	393.1150	393.1191	-10.43	$C_{19}H_{22}O_{9}$	2	10-hydroxyoleuropein aglycone isomer 2	137/181	(Serrano-García et al., 2022)
59	12.15	377.1266	377.1242	6.36	$C_{19}H_{22}O_{8}$	2	Oleuropein aglycone isomer 3	95/139	HMDB0301749
60	12.38	377.1236	377.1242	-1.59	$C_{19}H_{22}O_{8}$	2	Oleuropein aglycone isomer 4	95/139	HMDB0301749
61	12.54	377.1225	377.1242	-4.51	$C_{19}H_{22}O_{8}$	2	Oleuropein aglycone isomer 5	95/139	HMDB0301749

62	12.58	377.1231	377.1242	-2.92	$C_{19}H_{22}O_8$	2	Oleuropein aglycone isomer 6	95/139	HMDB0301749
63	12.59	287.2218	287.2228	-3.48	$C_{16}H_{32}O_4$	2	Dihydroxyhexadecanoic acid	99/134	HMDB0037798
64	12.95	315.0487	315.0510	-7.30	$C_{16}H_{12}O_7$	3	Isorhamnetin isomer 1	-	Pubchem: 5281691
65	13.06	315.0492	315.0510	-5.71	$C_{16}H_{12}O_7$	3	Isorhamnetin isomer 2	-	Pubchem: 5281691
66	13.41	377.1241	377.1242	-0.27	$C_{19}H_{22}O_8$	2	Oleuropein aglycone isomer 7	95/139	HMDB0301749
67	13.47	391.1384	391.1398	-3.58	$C_{20}H_{24}O_8$	2	Methyl-oleuropein aglycone	179/211/151	(Garcia-Aloy et al., 2020)
68	13.53	899.2973	899.2979	-0.67	$C_{44}H_{52}O_{20}$	4	Unknown	-	-
69	13.58	307.1926	307.1915	3.58	$C_{18}H_{28}O_4$	2	Dihydrocapsiate	235/185	Pubchem: 9873754
70	13.61	513.1774	513.1766	1.56	$C_{27}H_{30}O_{10}$	2	Baohuoside I	173/217/366	HMDB0248860
71	13.71	659.2274	659.2345	-10.77	$C_{33}H_{40}O_{14}$	2	2′′-O-Rhamnosylicariside II	366	Pubchem: 5318987
72	13.92	293.1754	293.1758	-1.36	$C_{17}H_{26}O_4$	3	Phytuberin	-	(Sánchez-Martínez et al., 2022)
73	14.03	305.1746	305.1758	-3.93	$C_{18}H_{26}O_4$	3	Dioxooctadecatrienoic acid	-	Pubchem: 18546963
74	14.35	513.1798	513.1766	6.24	$C_{27}H_{30}O_{10}$	3	Icariin II	-	Pubchem: 123134739
75	15.00	721.3721	721.3746	-3.47	$C_{45}H_{54}O_8$	4	Unknown	-	-
76	15.21	485.3277	485.3272	1.03	$C_{30}H_{46}O_5$	2	Quillaic acid	439/409	Pubchem: 101810

77	15.46	647.3255	647.3226	4.48	$C_{38}H_{48}O_9$	3	8,8a-Dihydro-8-hydroxygambogenic acid	-	(Tao et al., 2009)
78	15.74	293.2135	293.2122	4.43	$C_{18}H_{30}O_3$	2	Hydroxylinolenic acid isomer 1	275/235	HMDB0011108
79	15.80	293.2120	293.2122	-0.68	$C_{18}H_{30}O_3$	2	Hydroxylinolenic acid isomer 2	275/235	HMDB0011108
80	15.84	293.2115	293.2122	-2.39	$C_{18}H_{30}O_3$	2	Hydroxylinolenic acid isomer 3	275/235	HMDB0011108
81	16.03	487.2899	487.2913	-2.87	$C_{25}H_{44}O_9$	3	Lankolide	-	Pubchem: 11465948
82	16.30	540.3291	-	-	-	4	Unknown	-	-
83	16.42	295.2278	295.2279	-0.34	$C_{18}H_{32}O_3$	3	Hydroxylinoleic acid	-	HMDB0247599
84	16.60	566.3530	566.3460	12.36	$C_{31}H_{51}O_9$	4	Unknown	-	-
85	16.97	469.3303	469.3323	-4.26	$C_{30}H_{46}O_4$	3	Glycyrrhetinic acid	-	Pubchem: 73398
86	17.21	471.3464	471.3480	-3.39	$C_{30}H_{48}O_4$	3	Maslinic acid	-	HMDB0002392
87	17.55	799.3973	799.3910	7.88	$C_{43}H_{60}O_{14}$	2	Oleuropein derivative (oleuropein + C18H28O)	539/277/307/377	(Contreras et al., 2021)
88	18.46	277.2167	277.2173	-2.16	$C_{18}H_{30}O_2$	3	Linolenic acid	-	HMDB0001388
89	18.58	777.4053	777.4067	-1.80	$C_{41}H_{62}O_{14}$	2	Oleuropein derivative (oleuropein + C16H30O)	539/275/307/377	(Contreras et al., 2021)
90	18.73	375.2712	375.2693	5.06	$C_{27}H_{36}O$	2	10'-Apo-beta-carotenal	116	HMDB0059605
91	19.00	617.3837	617.3848	-1.78	$C_{39}H_{54}O_6$	3	3-beta-O-(trans-p-Coumaroyl)maslinic acid	-	Pubchem: 14335962
92	19.16	279.2328	279.2330	-0.72	$C_{18}H_{32}O_2$	3	Linoleic acid	-	HMDB0000673
93	19.64	933.6920	-	-	-	4	Unknown	-	-
94	19.82	255.2325	255.2330	-1.96	$C_{16}H_{32}O_2$	2	Palmitic acid	190/110	HMDB0000220

95	19.85	403.3052	403.3065	-3.22	$C_{22}H_{44}O_6$	3	Hexadecyl D-glucoside	-	Pubchem: 171356
96	19.95	281.2482	281.2486	-1.42	$C_{18}H_{34}O_2$	2	Oleic Acid	211/101	HMDB0000207
97	20.30	391.2838	391.2854	-4.09	$C_{24}H_{40}O_4$	4	Unknown	-	-
98	20.39	621.2719	621.2705	2.25	$C_{35}H_{42}O_{10}$	3	1-Hydroxy taxuspine C	-	Pubchem: 15923424
99	20.97	383.1916	383.1923	-1.83	$C_{16}H_{32}O_{10}$	3	Hexanedioic acid derivative	-	Pubchem: 88032455
100	21.24	758.5444	-	-	-	4	Unknown	-	-

RT: Retention Time; Mol. Formula: Molecular Formula

Table S6. Numerical results (mean and SD) and statistics significance for Figure 1 results.

	Cell viability (%)														
	HS			LC			OE			SM			TC		
**Conc. (µg/mL)**	**Mean**	**SD**	**Sign.**	**Mean**	**SD**	**Sign.**	**Mean**	**SD**	**Sign.**	**Mean**	**SD**	**Sign.**	**Mean**	**SD**	**Sign.**
**0**	100.00	2.41		100.00	1.92		100.00	3.75		100.00	4.04		100.00	2.25	
**5**	100.19	2.09	ns	106.16	1.92	ns	97.30	4.02	ns	99.31	4.03	ns	97.47	3.62	ns
**10**	97.37	5.69	ns	105.84	5.54	ns	96.71	3.60	ns	99.36	4.69	ns	101.09	2.62	ns
**15**	103.05	1.87	ns	109.53	4.59	***	96.04	4.41	ns	97.40	5.00	ns	101.14	5.97	ns
**20**	99.77	1.30	ns	110.97	4.49	****	93.02	2.71	ns	96.18	4.06	ns	101.10	3.03	ns
**25**	103.70	2.71	ns	96.77	2.68	ns	96.81	2.99	ns	97.35	4.10	ns	98.46	1.04	ns
**30**	101.25	2.15	ns	95.82	1.29	ns	94.06	4.12	ns	95.38	2.93	ns	100.30	3.67	ns
**40**	100.05	2.21	ns	98.48	1.99	ns	92.53	5.88	ns	95.74	3.42	ns	97.28	1.96	ns
**50**	97.57	2.83	ns	91.44	5.59	****	87.62	4.11	****	95.51	4.65	ns	99.41	2.51	ns
**60**	98.77	3.16	ns	87.56	2.78	****	81.51	4.19	****	90.24	2.59	**	98.03	2.30	ns
**80**	98.32	4.63	ns	69.95	1.77	****	71.72	7.37	****	91.84	5.65	*	98.11	3.60	ns
**100**	96.49	3.11	ns	68.24	1.99	****	68.43	4.10	****	87.71	3.33	****	103.62	2.97	ns
**150**	94.50	5.82	ns	51.30	2.74	****	37.77	6.16	****	72.20	3.60	****	103.84	3.41	ns
**200**	88.98	4.37	****	32.41	1.73	****	26.04	3.20	****	64.08	4.72	****	98.51	1.79	ns
**250**	85.61	1.41	****	28.39	2.35	****	25.26	1.50	****	67.01	2.21	****	105.05	2.22	ns
**300**	81.52	3.51	****	30.46	2.33	****	24.75	0.81	****	61.96	0.56	****	102.69	2.95	ns
**400**	68.47	1.75	****	31.05	1.60	****	25.75	1.18	****	50.24	1.12	****	94.96	3.91	ns
**500**	63.38	1.88	****	35.06	2.02	****	26.65	0.75	****	37.23	2.36	****	85.16	3.16	****
**600**	58.82	1.56	****	37.52	1.00	****	27.40	1.83	****	23.45	1.64	****	76.42	4.09	****

ns (non statistical significance), * ($p < 0.05$), ** ($p<0.01$), *** ($p<0.001$) and **** ($p<0.0001$).

Table S7. Equivalent DMSO concentrations for each extract concentration.

Equivalent extract conc. (ug/mL)	DMSO (%)	Cell viability (%)		
		Mean	SD	Sign.
**0**	**0**	100.00	5.65	ns
**300**	**0.3**	99.37	2.17	ns
**400**	**0.4**	99.78	8.00	ns
**500**	**0.5**	97.62	3.66	ns
**600**	**0.6**	98.16	4.17	ns
**800**	**0.8**	91.22	3.69	****

*ns (non statistical significance) and **** (p<0.0001).*
